# Plant A20/AN1 protein serves as the important hub to mediate antiviral immunity

**DOI:** 10.1371/journal.ppat.1007288

**Published:** 2018-09-13

**Authors:** Li Chang, Ho-Hsiung Chang, Jui-Che Chang, Hsiang-Chia Lu, Tan-Tung Wang, Duen-Wei Hsu, Yuh Tzean, An-Po Cheng, Yi-Shu Chiu, Hsin-Hung Yeh

**Affiliations:** 1 Agricultural Biotechnology Research Center, Academia Sinica, Taipei, Taiwan; 2 Department of Plant Pathology and Microbiology, National Taiwan University, Taipei, Taiwan; 3 Department of Biotechnology, National Kaohsiung Normal University, Kaohsiung, Taiwan; University of California, Davis Genome Center, UNITED STATES

## Abstract

Salicylic acid (SA) is a key phytohormone that mediates a broad spectrum of resistance against a diverse range of viruses; however, the downstream pathway of SA governed antiviral immune response remains largely to be explored. Here, we identified an orchid protein containing A20 and AN1 zinc finger domains, designated Pha13. Pha13 is up-regulated upon virus infection, and the transgenic monocot orchid and dicot *Arabidopsis* overexpressing orchid Pha13 conferred greater resistance to different viruses. In addition, our data showed that *Arabidopsis* homolog of *Pha13*, *AtSAP5*, is also involved in virus resistance. Pha13 and AtSAP5 are early induced by exogenous SA treatment, and participate in the expression of SA-mediated immune responsive genes, including the master regulator gene of plant immunity, *NPR1*, as well as NPR1-independent virus defense genes. SA also induced the proteasome degradation of Pha13. Functional domain analysis revealed that AN1 domain of Pha13 is involved in expression of orchid NPR1 through its AN1 domain, whereas dual A20/AN1 domains orchestrated the overall virus resistance. Subcellular localization analysis suggested that Pha13 can be found localized in the nucleus. Self-ubiquitination assay revealed that Pha13 confer E3 ligase activity, and the main E3 ligase activity was mapped to the A20 domain. Identification of Pha13 interacting proteins and substrate by yeast two-hybrid screening revealed mainly ubiquitin proteins. Further detailed biochemical analysis revealed that A20 domain of Pha13 binds to various polyubiquitin chains, suggesting that Pha13 may interact with multiple ubiquitinated proteins. Our findings revealed that Pha13 serves as an important regulatory hub in plant antiviral immunity, and uncover a delicate mode of immune regulation through the coordination of A20 and/or AN1 domains, as well as through the modulation of E3 ligase and ubiquitin chain binding activity of Pha13.

## Author summary

The Salicylic acid (SA)-mediated plant immunity plays a major role against diverse pathogens including viruses. However, the underlying SA-mediated virus resistance pathway is not fully understood. Here, we identified the A20 and AN1 zinc finger domain containing protein, Pha13, in *Phalaenopsis aphrodite*. We provided evidences that Pha13 and its *Arabidopsis* homolog, AtSAP5, mediates the conserved immune response against diverse viruses in both orchid and *Arabidopsis*. SA affects Pha13 on both transcriptional and post-translational levels. In addition, Pha13 and AtSAP5 is involved in the expression of well-known master regulator, NPR1, as well as NPR1-independent genes, and serve as a regulatory hub in the SA-mediated immune pathway. Biochemical analysis indicated that Pha13 confers both E3 ligase and ubiquitin binding activity, which mainly functions through the A20 domain. We also showed that Pha13 has the ability to bind various ubiquitin chains, suggesting that Pha13 may interact to multiple ubiquitinated substrates for the regulation of immune genes. In summary, Pha13 and AtSAP5 function in the previously unknown SA-mediated antiviral immune pathway. The diversified biochemical properties and distinct function of A20/AN1 domains revealed the sophisticated regulation of Pha13 in antiviral immunity.

## Introduction

The plant hormone salicylic acid (SA) plays a major role in triggering local and systemic immune response for combating a broad-spectrum of biotrophic pathogens including viruses [1–3]. SA is involved in innate immunity including pattern-triggered immunity (PTI) and effector-triggered immunity (ETI) to ward off invaders [1]. Plants trigger PTI as the first line of defense through recognition of conserved microbe-associated molecular patterns (MAMPs) by pattern-recognition receptors [4, 5]. PTI is also involved in plant viral resistance, and virus dsRNA has been shown to serve as a MAMP [6–8]. To successfully infect plant hosts, pathogens utilize various effectors to compromise PTI [9]. However, plants have evolved resistance (R) proteins capable of detecting these effectors to trigger ETI, which is a second line of plant defense against viruses [9]. Of note, elevated SA concentration is also essential to establish systemic acquired resistance (SAR) to further protect plants from diverse pathogens [10]. In summary, SA plays an important role in the regulation of PTI, ETI, and SAR to ward off virus infection [1].

The importance of SA-induced immunity has led to multiple screens for genes involved in the SA signaling pathway using pathogenesis-related protein (PR) genes as markers. However, various unrelated studies have only identified a single genetic locus, *npr1* [11–13]. NPR1 is conserved among plants and is a master regulator of the SA-induced plant immune signaling pathway [10]. *NPR1* transcription is moderately induced (2–3 times) upon pathogen or SA treatment [14, 15], and the post-translational regulation of NPR1 is essential to trigger immune response [16]. Even though plant NPR1 is seen to play important roles in regulating SA-induced plant immunity, SA-induced virus resistance still occurs in *Arabidopsis npr1* mutants [17, 18], leading to questions about SA-induced NPR1-independent virus defense.

In addition to PTI and ETI, gene silencing is also an important defense mechanism for combating viruses [19]. It is triggered by the presence of viral dsRNA, which is processed to small-interfering dsRNA (siRNA) by Dicer-like nuclease. The RNA dependent RNA polymerase (RdR1) is responsible for the *de novo* synthesis of dsRNA to initiate secondary RNA silencing against viruses in plants [20–22]. RdR1 can be up-regulated after SA treatment [23], and it is also dependent on NPR1 in *Arabidopsis* [24]. Yet, RdR1 may not completely resolve the mechanism of SA-induced virus defense since SA treatment can still trigger resistance to *Tobacco mosaic virus* in *Nicotiana benthamiana* with non-functional RdR1 [25].

Studies on diverse plants enable a broader understanding of comprehensive strategies applied by plants to cope with stresses. The Orchidaceae is among one of the largest family of flowering plants [26]; however, the slow growth and difficulty in regeneration has hampered the study of orchids. In order to facilitate gene functional studies in orchid, we previously developed a high-throughput *Cymbidium mosaic virus*-induced gene silencing system (CymMV-VIGS) [27]. Here, through VIGS screening of immunity related genes in orchids, we identified a regulator, Pha13, which is involved in the SA immune signaling pathway. Transgenic monocot orchid and dicot *Arabidopsis* plants overexpressing Pha13 showed greatly enhanced resistance to different viruses. Transgenic *Arabidopsis* overexpressing Pha13 also enhanced plant resistance to *Pseudomonas syringae* pv. *tomato* DC30000. Our detailed analysis revealed that Pha13 is regulated by SA and leads to transcriptional reprograming of massive numbers of immune responsive genes including the master regulator gene of plant immunity, *NPR1*, as well as NPR1-independent virus defense genes. The AN1 domain was shown to be associated to the expression of orchid NPR1, and both A20 and AN1 domains are required for virus resistance. Pha13 exhibits similar and distinctive biochemical features to other A20/AN1 proteins, including domains involved in E3 ligase and polyubiquitin chain binding activity, with known A20/AN1 proteins. Our findings revealed that Pha13 is conserved in mediating viral resistance among plants and serves as a pivotal regulatory hub in antiviral immunity.

## Results

### *Pha13* is involved in expression of SA-induced *PhaNPR1*

**Fig 1:**
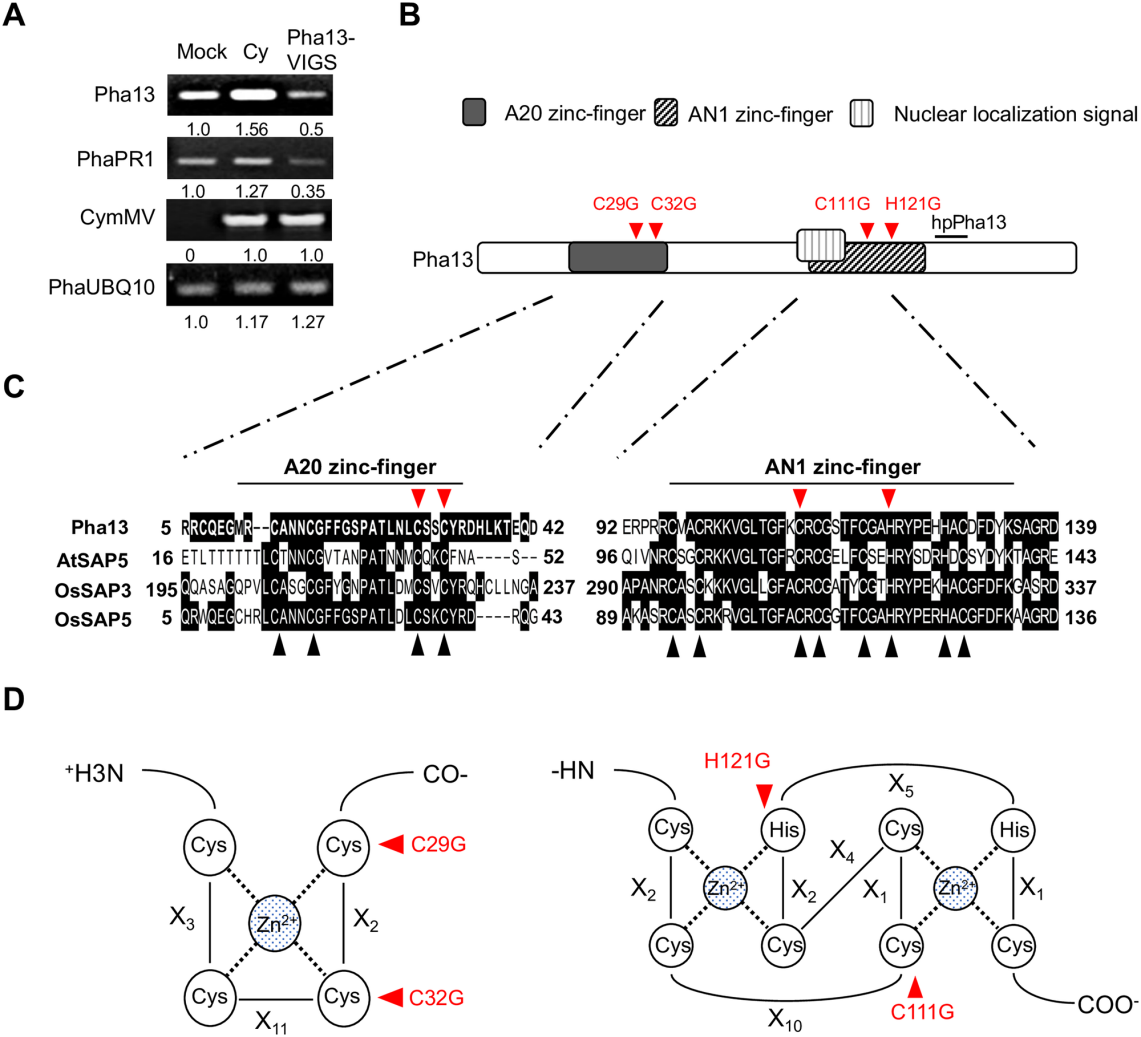
Identification of Pha13 involvement in *PhaPR1* expression by CymMV-based VIGS system and sequence analysis. (A) Expression level of *Pha13*, *PhaPR1*, and CymMV were analyzed by RT-PCR from leaves of *P*. *aphrodite* inoculated with buffer (Mock), or infiltrated with agrobacterium carrying pCymMV (Cy) or pCymMV-Pha13 (Pha13-VIGS). *PhaUbiquitin 10* (PhaUBQ10) was used as a loading control, and the relative expression level of corresponding genes are indicated. (B) Domains of Pha13. Open rectangle indicates the entire protein. A20 (black rectangle) and AN1 (diagonally-striped rectangle), nuclear localization signal (open square with vertical lines), and 21-nucleotide position (short black line) used for designing hairpin RNA of *Pha13* (hpPha13) are indicated. (C) Sequence alignment of A20/AN1 zinc finger domains of Pha13 with stress-associated proteins from *Arabidopsis thaliana* (AtSAP5, accession number: AT3G12630), and *Oryza sativa* (OsSAP3, accession number: LOC_Os01g56040.1; OsSAP5, accession number: LOC_Os02g32840.1) are shown. Conserved amino acid sequences are indicated with a black box. Conserved cysteine (C) and histidine (H) are indicated with a black triangle. (D) Primary sequence organization of A20 and AN1 domains. Xn: the number of amino acid residues between zinc ligands. (B to D) The mutation positions for domain functional analysis are indicated with red triangle.

*Pha13* (Orchidstra 2.0 database, http://orchidstra2.abrc.sinica.edu.tw, accession number PATC148746, the Pha13 coding sequence can be found in S1 File) was identified here for the first time using the CymMV-VIGS system in *Phalaenopsis aphrodite*. Our data indicated that silencing *Pha13* decreased the RNA of *PhaPR1* (Fig 1A). Amino acid sequence analysis revealed that Pha13 contained A20 and AN1 domains (Fig 1B–1D) and belonged to a fast emerging class of zinc-finger proteins (ZFPs), termed stress associated proteins (SAPs) in plants [28].

**Fig 2:**
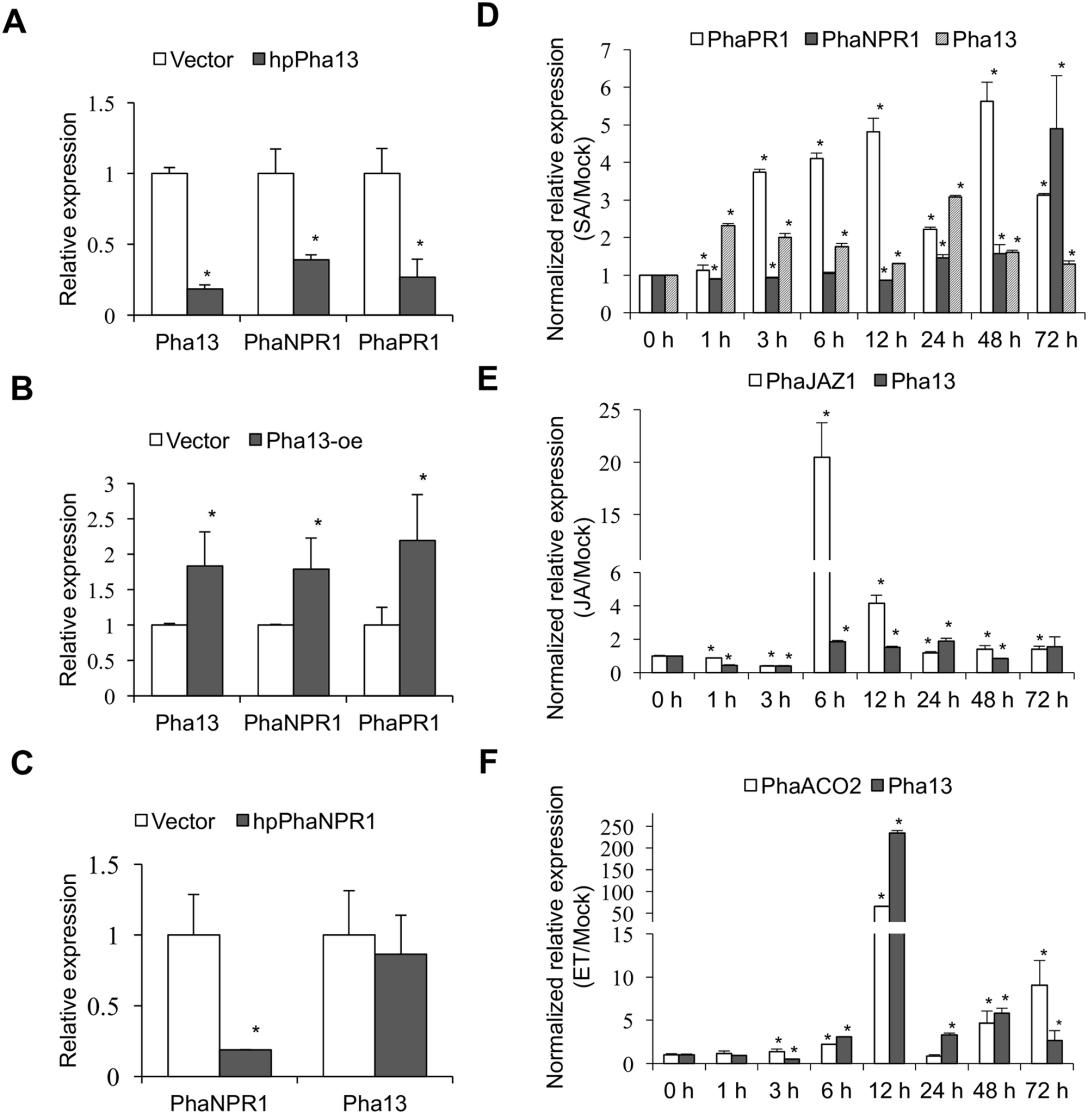
*Pha13* is involved in the expression of *PhaPR1* and *PhaNPR1*, and induced by phytohormone treatment. (A and B) Expression level of *Pha13*, *PhaNPR1*, and *PhaPR1* were analyzed by qRT-PCR from leaves of *P*. *aphrodite* infiltrated with agrobacterium carrying vector (Vector); hairpin RNA (hpRNA) vector to knock down *Pha13* (hpPha13; A); and overexpression vector of *Pha13* (Pha13-oe; B). The RNA level of vector was set to 1. (C) Transient silencing of *PhaNPR1*. Expression level of *PhaNPR1* and *Pha13* were analyzed by qRT-PCR from leaves of *P*. *aphrodite* infiltrated with agrobacterium carrying the vector (Vector) or hairpin RNA (hpRNA) vector to knock down *PhaNPR1* (hpPhaNPR1). The RNA level of the vector was set to 1. (A to C) Mean ± SD; n = 3 biological replicates; *, *P* < 0.05, Student’s t-test compared to vector. (D to F) Time-course expression of *Pha13* under different plant hormone treatments in *P*. *aphrodite*. Expression level of *Pha13* was analyzed by qRT-PCR from leaves treated with SA (D), JA (E), and ET (F) at different hours (h) post-treatment. Inoculation buffer treatment was used as a mock control. Results of qRT-PCR were relative to that of mock at individual time course for relative quantification. The RNA level at 0 hour was set to 1 for comparison between different time courses. *PhaPR1* and *PhaNPR1* were used as SA marker genes. *PhaJAZ1* and *PhaACO2* were used as JA and ET marker genes, respectively. Data represent mean ± SD; n = 3 technical replicates; *, *P* < 0.05, Student’s t-test compared to 0 h. One representative experiment is shown from at least three replicates of similar results. *PhaUbiquitin 10* was used as an internal control for normalization.

To further address the interrelationship between Pha13 and known SA-related genes, we knocked down *Pha13* RNAs by infiltration of agrobacterium carrying the 35S promoter driven hairpin RNA-expressing constructs of phpPha13 in *P*. *aphrodite* (Fig 2A). Total RNAs extracted from agrobacterium-infiltrated plants were used to detect RNA expression of *Pha13*, *PhaNPR1*, and *PhaPR1* by qRT-PCR. Knockdown of *Pha13* RNA is correlated with decreased RNA levels of *PhaNPR1* and *PhaPR1* (Fig 2A). Furthermore, we generated transient overexpression of Pha13 in *P*. *aphrodite* by infiltration of agrobacterium carrying the overexpression constructs (pPha13-oe). Transient overexpression of *Pha13* in *P*. *aphrodite* increased the RNA levels of *PhaNPR1* and *PhaPR1* (Fig 2B). On the other hand, transient silencing of *PhaNPR1* (infiltration of agrobacterium carrying phpPhaNPR1) did not affect the RNA accumulation of *Pha13* (Fig 2C). This indicates that Pha13 affects the expression of *PhaNPR1* but not vice versa.

The results from our transient silencing and overexpression of Pha13 showed that Pha13 is involved in the SA-related plant immune pathway. Next, we tested whether SA or other plant hormones affect *Pha13* transcription. Plant samples were collected at different time points up to 72 h after SA, jasmonic acid (JA), and ethylene (ET) treatment, and analyzed for RNA of Pha13 and corresponding hormone-marker genes by qRT-PCR. Pha13 was induced by SA, JA, and ET at 1 h, 6 h, and 6 h post-treatment, respectively (Fig 2D–2F). The results indicated that post-treatment expression of Pha13 was induced earlier in SA treatment, at 1 h post-treatment, compared to up regulation of *PhaNPR1* at 24 h post-treatment (Fig 2D).

### *Pha13* is induced by CymMV infection and involved in virus resistance

**Fig 3:**
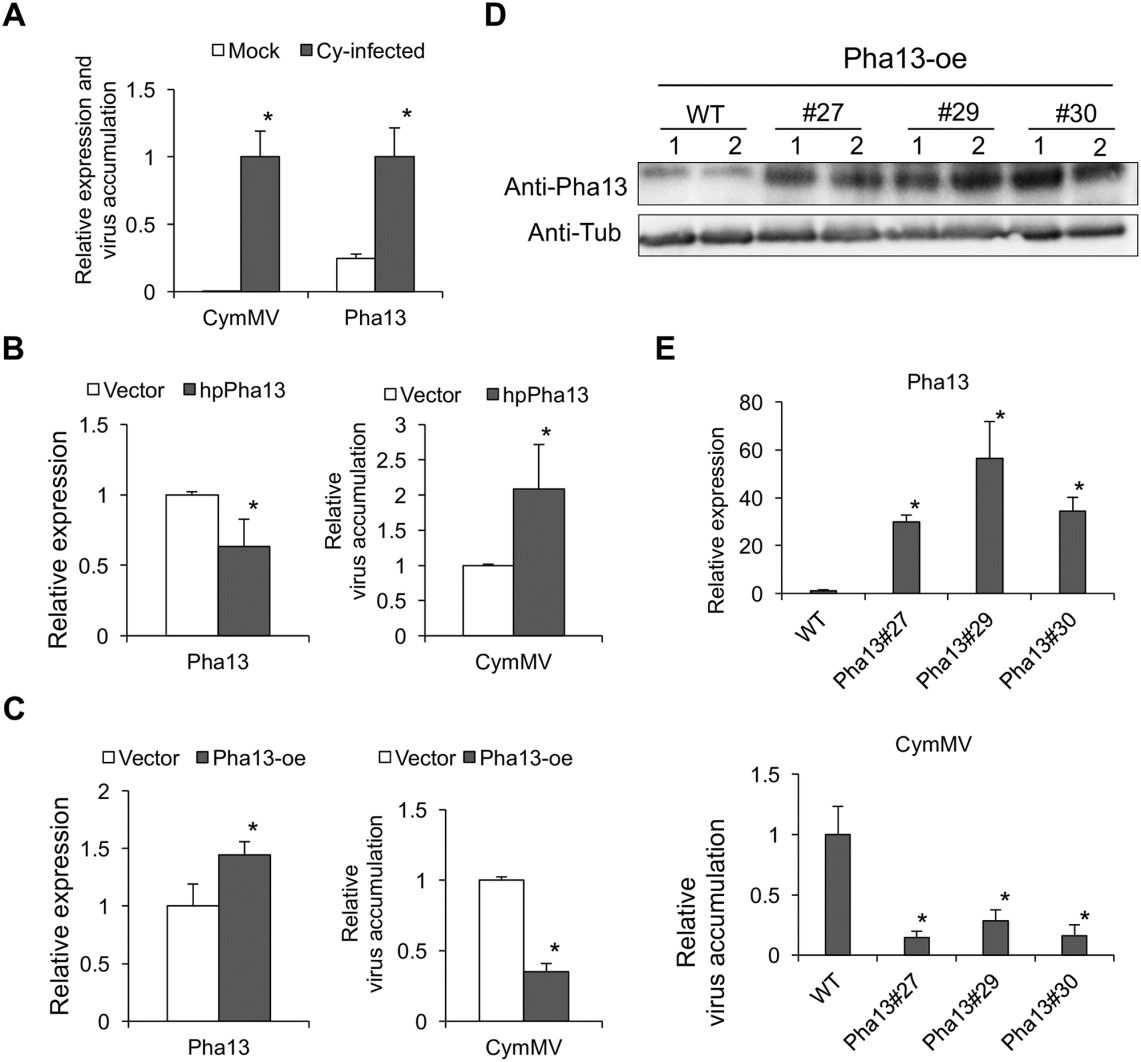
*Pha13* is induced by CymMV and involved in virus accumulation. (A) The CymMV accumulation level and expression level of *Pha13* were analyzed by qRT-PCR in healthy (Mock) and CymMV-infected (Cy-infected) *P*. *aphrodite*. The RNA level in the CymMV was set to 1. Data represent mean ± SD; n = 3 biological replicates; *, *P* < 0.05, Student’s t-test compared to mock. (B and C) Transient silencing or overexpression of Pha13 in CymMV-infected plants. RNA level of *Pha13* and CymMV were analyzed by qRT-PCR from leaves of CymMV-infected *P*. *aphrodite* infiltrated with agrobacterium carrying vector (Vector); hairpin RNA (hpRNA) vector to knockdown *Pha13* (hpPha13; B); or overexpression vector of Pha13 (Pha13-oe; C). The RNA level of vector was set to 1. Data represent mean ± SD; n = 3 biological replicates; *, *P* < 0.05, Student’s t-test compared to vector. D, The protein level of Pha13 in WT and transgenic *P*. *equestris* (Pha13#27, #29 and #30) expressing Pha13, Pha13-oe (35S::Flag-Pha13), were analyzed by anti-Pha13 antibody. The anti-Tubulin antibody (Anti-Tub) was used as a loading control. (E) Three wild-type plants and three plants derived from the same transformed protocorm were used for analysis. RNA level of *Pha13* and CymMV were analyzed by qRT-PCR from leaves of WT or transgenic *P*. *equestris* (Pha13#27, #29 and #30) inoculated with CymMV. The RNA level of WT was set to 1. Data represent mean ± SD; n = 3 biological replicates; *, *P* < 0.05, Student’s t-test compared to WT. *PhaUbiquitin 10* was used as an internal control for normalization.

To determine whether *Pha13* is involved in plant resistance to virus infection, we first analyzed *Pha13* expression in mock- or CymMV-inoculated *P*. *aphrodite* by qRT-PCR. The results showed that *Pha13* is induced by CymMV (Fig 3A). Furthermore, we assayed the virus accumulation in transient silenced or overexpressed *Pha13* in *P*. *aphrodite*. Transient knockdown of *Pha13* increased CymMV accumulation in infected *P*. *aphrodite* (Fig 3B); overexpression of *Pha13* in CymMV-infected *P*. *aphrodite* decreased CymMV accumulation (Fig 3C). To validate the results, we generated transgenic *P*. *equestris* overexpressing Pha13 (35S::FLAG-Pha13). Western blot using antiserum against Pha13 indicated that the overexpressed Pha13 could be detected in every individual asexually propagated progeny derived from transgenic T0 lines 27, 29 and 30 (Fig 3D). When we inoculated CymMV into 3 individual progenies derived from 3 transgenic lines, the results showed that CymMV decreased to a very low level in all progenies of the three transgenic lines as compared to the non-transgenic lines (Fig 3E).

### Pha13 participates in the expression of SA-induced PhaNPR1-dependent and -independent virus resistance

To explore the gene(s) affected by Pha13, we conducted microarray analysis of *P*. *aphrodite* overexpressing Pha13 (pPha13-oe). Global gene expression analysis comparing microarray data of plants overexpressing Pha13 to the vector control 5 days post agroinfiltration revealed that overexpression of Pha13 affected the expression of 10639 genes (S1A Fig). Gene ontology of the affected genes indicated that Pha13 is involved in cellular processes, metabolic processes, and single-organism processes (S1B Fig). To verify the microarray data, we analyzed the RNA expression level by qRT-PCR of three genes *PhaNPR1*, *PhaRdR1*, and *Glutaredoxin* (*PhaGRX*), that were up-regulated in the Pha13 overexpression plants (S2 Fig).

In our analysis of differentially expressed genes of plants overexpressing Pha13, we observed several genes previously reported to be involved in plant resistance. Among them are SA-induced genes that are known to be NPR1-dependent and -independent (S1 Table). We selected two genes, *PhaRdR1* and *PhaGRX*, reported to be involved in plant resistance [29, 30] and induced by SA, for further analysis. One of the selected genes, *RdR1* is positively regulated by *NPR1* [24], whereas the regulation of *GRX* is irrelevant to *NPR1* in *Arabidopsis* [31].

**Fig 4:**
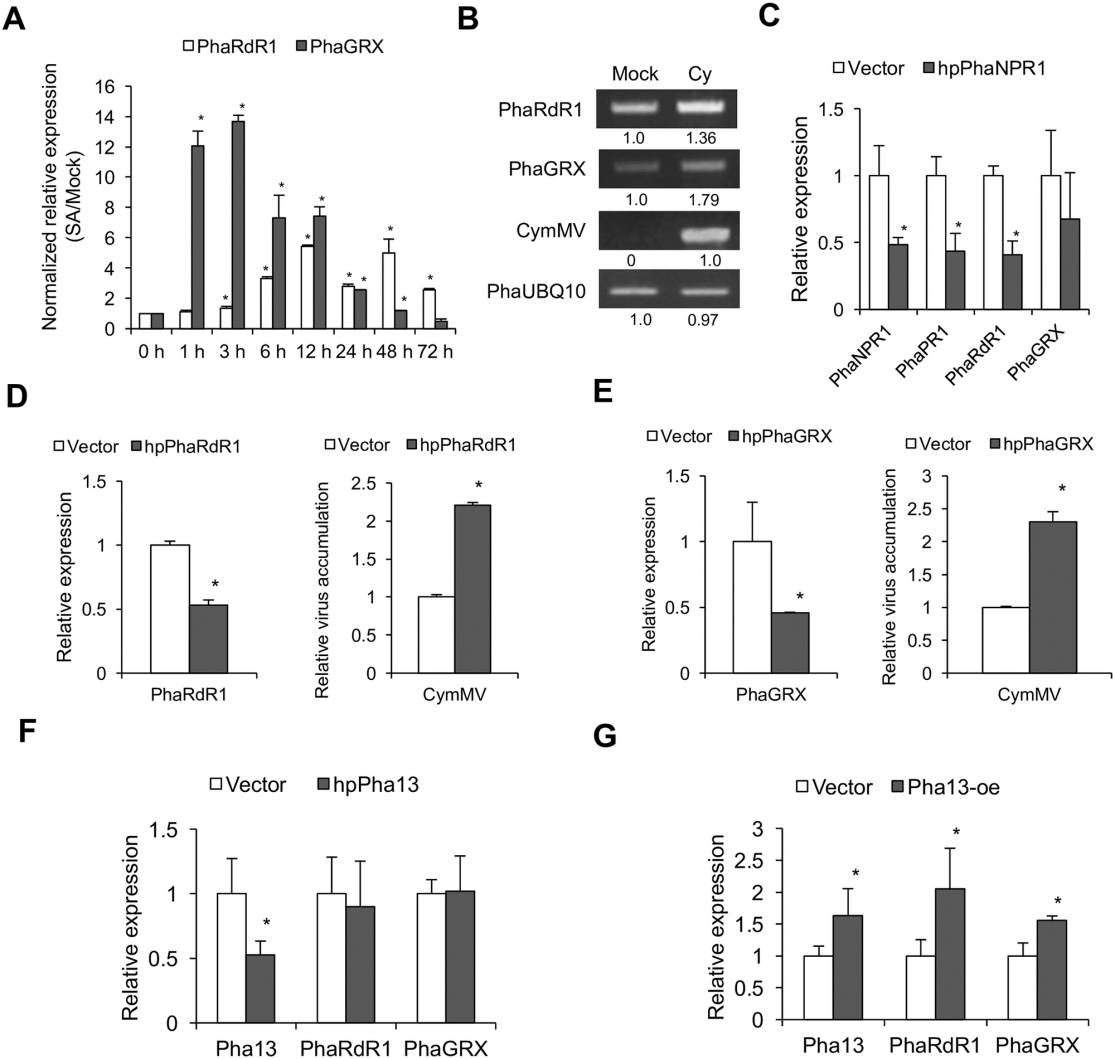
*Pha13* is involved in the expression of *PhaNPR1*-dependent and -independent marker genes. (A) Expression level of *PhaRdR1* and *PhaGRX* were analyzed by qRT-PCR from leaves of *P*. *aphrodite* treated with buffer (Mock) or SA, at different times (h) post-treatment. Results of qRT-PCR were relative to that of mock at individual time course for relative quantification. The RNA level at 0 h was set to 1. Data represent mean ± SD; n = 3 technical replicates; *, *P* < 0.05, Student’s t-test compared to 0 h. One representative experiment is shown from at least three replicates of similar results. *PhaUbiquitin 10* was used as an internal control for normalization. (B) Expression level of *PhaRdR1*, *PhaGRX*, and CymMV accumulation level were analyzed by semi-quantitative RT-PCR from leaves of *P*. *aphrodite* inoculated with buffer (Mock) or infected with CymMV (Cy). *PhaUbiquitin 10* was used as a loading control, and relative expression levels of the corresponding genes are indicated. (C) Expression level of *PhaNPR1*, *PhaPR1*, *PhaRdR1*, and *PhaGRX* were analyzed by qRT-PCR from leaves of *P*. *aphrodite* infiltrated with agrobacterium carrying the vector (Vector) or hairpin RNA (hpRNA) vector to knock down *PhaNPR1* (hpPhaNPR1). (D and E) Expression level of *PhaRdR1*, *PhaGRX*, and CymMV accumulation level were analyzed by qRT-PCR from leaves of CymMV-infected *P*. *aphrodite* infiltrated with agrobacterium carrying vector (Vector); hairpin RNA (hpRNA) vector to knockdown *PhaRdR1* (hpPhaRdR1, D), or *PhaGRX* (hpPhaGRX, E). (F and G) Expression level of *Pha13*, *PhaRdR1*, and *PhaGRX* were analyzed by qRT-PCR from leaves of *P*. *aphrodite* infiltrated with agrobacterium carrying vector (Vector) or hairpin RNA (hpRNA) vector to knockdown *Pha13* (hpPha13; F), or to overexpress Pha13 (Pha13-oe; G). C to G, The RNA level of vector was set to 1. Data represent mean ± SD; n = 3 biological replicates; *, *P* < 0.05, Student’s t-test compared to vector. *PhaUbiquitin 10* was used as an internal control for normalization.

In orchids, *PhaRdR1* and *PhaGRX* were both induced by SA and CymMV infection (Fig 4A and 4B). We next investigated whether *PhaNPR1* regulates *PhaRdR1* and *PhaGRX* expression in *P*. *aphrodite* through transiently delivering *PhaNPR1* hairpin RNA into *P*. *aphrodite* by agroinfiltration carrying phpPhaNPR1. Our data for orchids is consistent with studies in other systems, in which transient silencing of *PhaNPR1* can decrease the downstream marker genes, *PhaPR1* and *PhaRdR1*, whereas *PhaGRX* remain unchanged (Fig 4C).

To reveal whether *PhaRdR1* or *PhaGRX* is involved in virus resistance, we transiently silenced the two genes in CymMV pre-infected *P*. *aphrodite*. Consistent with previous reports [29], our data indicated that silencing *PhaRdR1* (phpPhaRdR1) increases CymMV accumulation (Fig 4D). Although a previous report indicated that *GRX* is involved in plant defense against *Botrytis cinerea* infection [30], its role in virus infection remains elusive. Our data indicated that silencing *PhaGRX* (phpPhaGRX) increased CymMV accumulation (Fig 4E).

Our data suggested that both *PhaRdR1* and *PhaGRX* are involved in SA-induced virus resistance. To further analyze the relationship between *Pha13* in the regulation of *PhaRdR1* and *PhaGRX*, we performed transient silencing and overexpression assay of Pha13 in *P*. *aphrodite*. Transient silencing *Pha13* did not affect the expression of *PhaRdR1* or *PhaGRX*, while transiently overexpressing Pha13 increased the expression of *PhaRdR1* and *PhaGRX* (Fig 4F and 4G). Our data suggested that *Pha13* positively regulates *PhaRdR1* and *PhaGRX* expression.

### Pha13 AN1 domain is involved in the expression of *PhaNPR1*, and A20 and AN1 domains are both required for induced expression of *PhaRdR1*, *PhaGRX*, and CymMV accumulation

**Fig 5:**
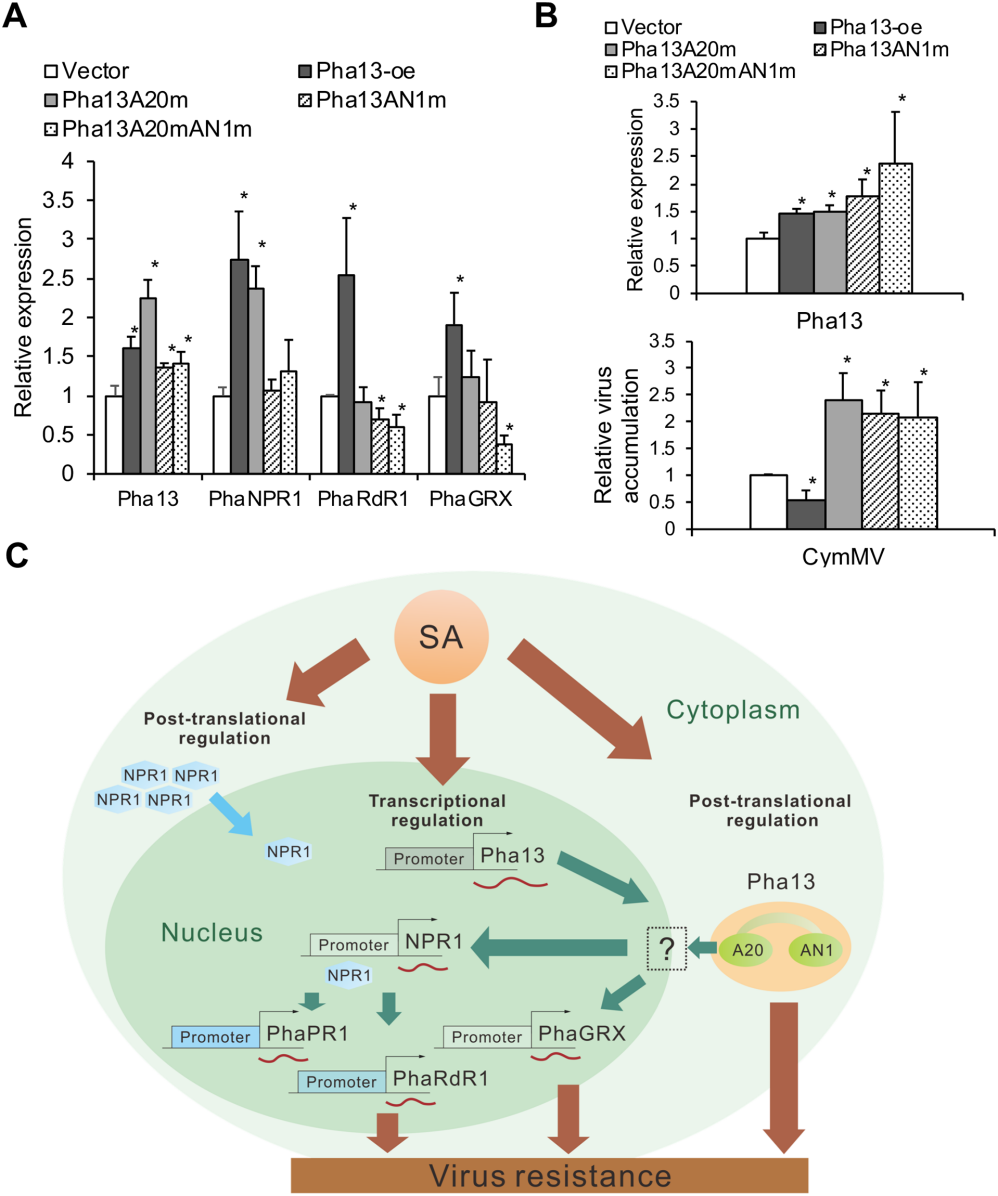
Pha13 A20 and AN1 domains are involved in the expression of PhaNPR1-dependent and -independent immune gene(s) and virus accumulation. (A and B) Expression level of *Pha13*, *PhaNPR1*, *PhaRdR1*, *PhaGRX*, and CymMV accumulation level were analyzed by qRT-PCR of healthy *P*. *aphrodite* leaves (A), or CymMV pre-infected *P*. *aphrodite* (B) and infiltrated with agrobacterium carrying vector (Vector), overexpression clones of Pha13, or the respective A20 and/or AN1 mutant clones. The RNA level of vector was set to 1. Data represent mean ± SD; n = 3 biological replicates; *, *P* < 0.05, Student’s t-test compared to vector. *PhaUbiquitin 10* was used as an internal control for normalization. (C) A model illustrating SA-induced *Pha13* transcriptional and post-translational regulation leading to the activation of immune responsive genes. Virus infection caused accumulation of SA and leads to post-translational modification of NPR1, allowing it to enter into the nucleus for the activation of NPR1-dependent immune responsive genes including *PR1* and *RdR1*. On the other hand, increased SA can also regulate *Pha13* at both transcriptional and post-translational level and leads to the expression of NPR1-dependent and independent immune responsive genes including *NPR1*, *RdR1* and *GRX* for virus resistance.

To analyze the function of A20 and AN1 domain in Pha13, we overexpressed wild-type or mutant of Pha13 (mutated A20, and/or AN1 domain of Pha13) in healthy or CymMV pre-infected *P*. *aphrodite*. We substituted the conserved cysteine and histidine to glycine on A20 and/or AN1 of Pha13 (Fig 1C and 1D) to generate the A20 domain mutant (Pha13A20m), AN1 domain mutant (Pha13AN1m), and the A20 and AN1 domain double mutant (Pha13A20mAN1m). The results indicated that overexpression of Pha13 A20 mutant (Pha13A20m) increased expression of *PhaNPR1*, which is consistent with overexpression of Pha13 wild-type (Fig 5A). The stable expression of Pha13 wild-type and mutants were confirmed by immunoblotting analysis (S3 Fig). Overexpression of Pha13 AN1 mutant (Pha13AN1m) in *P*. *aphrodite* decreased the expression of *PhaRdR1*, whereas the expression of *PhaNPR1* and *PhaGRX* remained unchanged (Fig 5A). Overexpression of Pha13 A20 and AN1 mutant (Pha13A20mAN1m) in *P*. *aphrodite* decreased the RNA levels of *PhaRdR1* and *PhaGRX*, while the expression of *PhaNPR1* remains unchanged (Fig 5A). Overexpression of any Pha13 A20 and/or AN1 mutant resulted in increased accumulation of CymMV (Fig 5B). These data are summarized in Fig 5C and S4 Fig. Our data suggests that Pha13 AN1 domain alone can affect the expression of *PhaNPR1*, and both A20 and AN1 domains are required for regulation of *PhaRdR1*, *PhaGRX*, and CymMV accumulation.

### Pha13 contains self-ubiquitination E3 ligase activity and the A20 domain is more important in conferring E3 ligase activity

**Fig 6:**
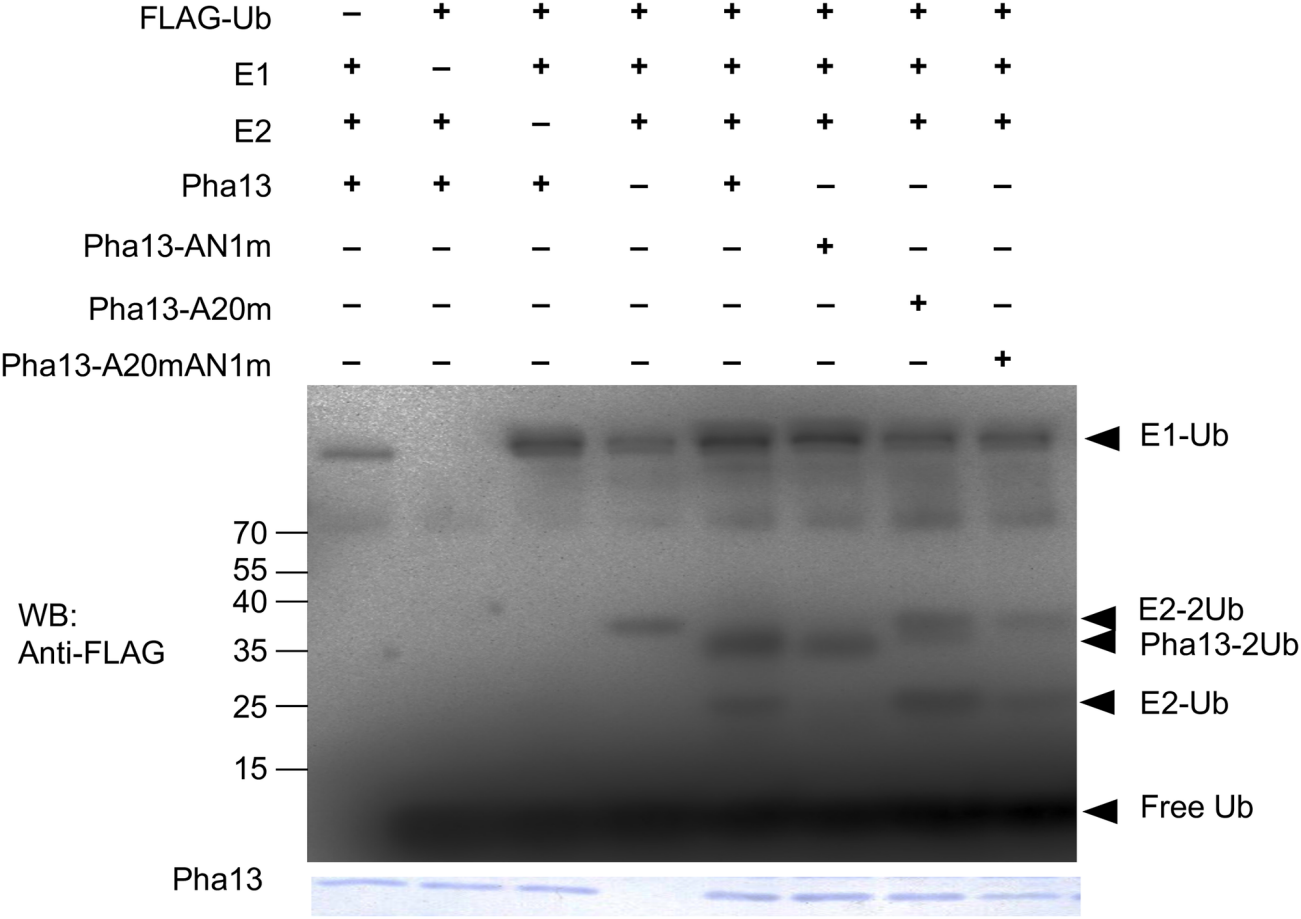
Pha13 confers self-ubiquitination E3 ligase and ubiquitin binding ability. In vitro ubiquitination analysis was performed on recombinant proteins of Pha13, A20 mutant (Pha13A20m), AN1 mutant (Pha13AN1m), A20/AN1 double mutant (Pha13A20mAN1m) with or without FLAG-tagged ubiquitin (FLAG-Ub), ubiquitin-activating enzyme (E1), or ubiquitin-conjugating enzyme (E2). Ubiquitinated proteins were analyzed with immunoblotting using anti-FLAG antibodies. Coomassie staining was used as a loading control. The ubiquitinated Pha13 (Pha13-2Ub), E1 conjugated with one Ub (E1-Ub), E2 conjugated with one or two Ub (E2-Ub, E2-2Ub), and free Ub are indicated with arrow.

A20-type zinc finger proteins have been reported to confer ubiquitin ligase activity [32]. Therefore, we analyzed whether Pha13 has self-ubiquitination E3 ligase activity. We purified His-tagged recombinant Pha13 (Pha13-His) from *E*. *coli* for self-ubiquitination E3 ligase activity analysis. Self-ubiquitination E3 ligase activity was observed in the presence of human E1 and E2 with Pha13-His (Fig 6) by using anti-FLAG antibody to detect FLAG-ubiquitin. Furthermore, we also analyzed the self-ubiquitination E3 ligase activity of the A20 and/or AN1 domain. As shown in Fig 6, E3 ligase activity of Pha13 was greatly reduced in A20 mutant (Pha13A20m) or A20/AN1 double mutant (Pha13A20mAN1m). AN1 mutant (Pha13AN1m) showed higher self-ubiquitination E3 ligase activity than other domain mutants, but lower than the wild-type form of Pha13 (Fig 6). The results indicate that A20 domain of Pha13 plays a more important role in conferring E3 ligase activity. The data are summarized in S4 Fig.

### Pha13 exhibited ubiquitin binding activity

**Fig 7:**
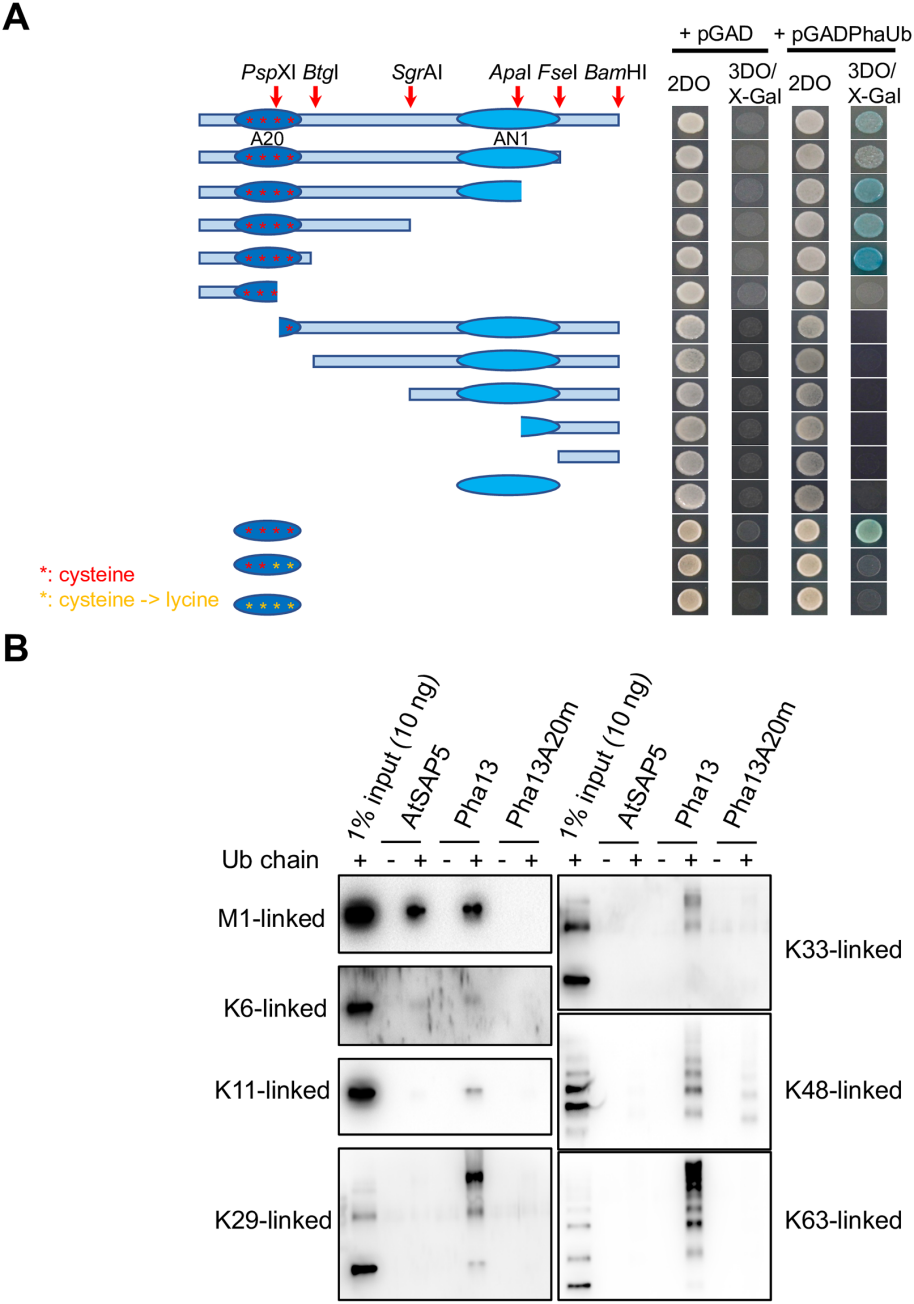
Mapping the ubiquitin-binding region in Pha13 by a yeast two-hybrid assay. (A) Left panel indicating Pha13 putative functional domains (A20 and AN1) and the sites of truncation. The ubiquitin-binding region is mapped to the A20 domain of Pha13. Various combinations of Pha13 truncations cloned in pGBK vector were co-transformed with pGADPhaUb into yeast AH109 strain. The transformants were spotted on control plates (2DO: Leucine-Trptophan dropped out medium) and selective plates (3DO/X-Gal: Leucine-Trptophan-Histidine dropped out and X-Gal added medium), which were incubated at 30°C for 4 days before photography. Red stars indicate cysteine in A20 domain. Yellow stars indicate cysteine replaced with lysine. (B) In vitro pull-down assay on AtSAP5 (used as positive control) [44], Pha13 and Pha13 A20 mutant (Pha13A20m), with polyubiquitin chains.

In addition, to search for Pha13 substrate or interacting proteins, we constructed a yeast two-hybrid (Y2H) library with RNA extracted from SA-treated *P*. *aphrodite*. In our initial screening, we identified 56 positive clones. After sequencing the clones, we found that the positive clones include 25 clones encoding ubiquitin, 4 clones encoding partial sequences of thioredoxin-like proteins, 3 clones encoding partial DNAJ-like proteins, and the rest of clones encode proteins of different identity that only appear once (S2 Table). The full length of clones encoding proteins appearing more than once in our initial screen were subjected to further Y2H analysis. Only clones encoding ubiquitin showed positive interaction (Fig 7A). To map the ubiquitin binding domain of Pha13, a series of pha13 deletion mutants were generated and used for Y2H assay (Fig 7A). The results indicated A20 domain is mainly involved in ubiquitin binding (Fig 7A).

To assay which type of ubiquitin chain binds to Pha13, in vitro pull-down assays were performed by mixing recombinant Pha13, A20 mutant (Pha13A20m), or AtSAP5 (positive control) with commercially available linear polyubiquitin chains. After precipitation, the bounded linear polyubiquitin was detected by immunoblot with the anti-ubiquitin antibody. As shown in Fig 7B, Pha13 has the ability to bind to M1, K6, K11, K29, K33, K48 and K63-linked ubiquitin chains, and A20 domain is mainly responsible for the binding.

### SA regulates Pha13 at post-translational level

**Fig 8:**
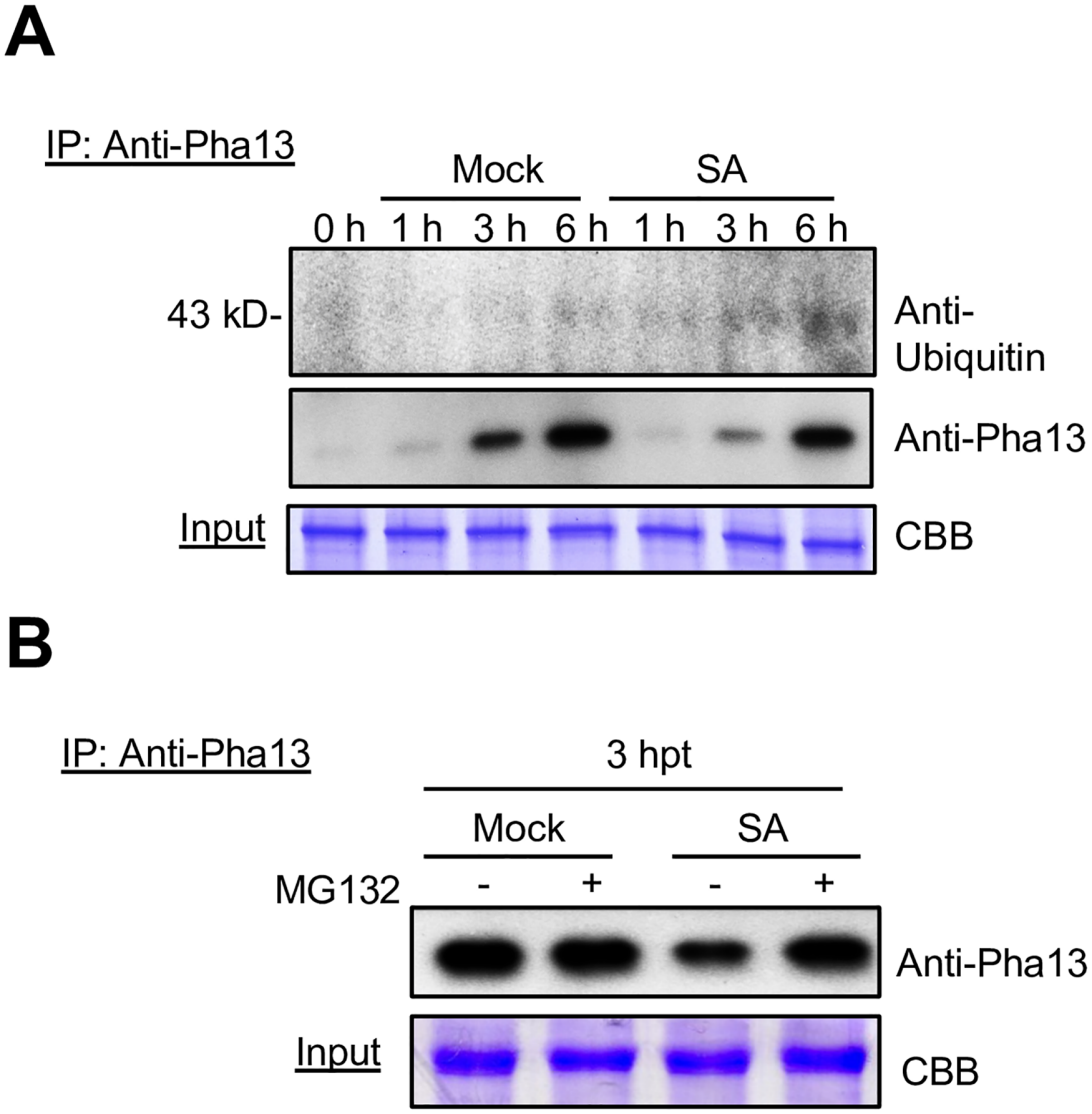
SA regulates Pha13 at post-translational level. (A) Total proteins were extracted from leaves of *P*. *aphrodite* without treatment (0 h), treated with H_2_O (Mock), or SA at different times (h), and used for in vivo immunoprecipitation (IP) assay using anti-Pha13 antibody. Samples after IP were analyzed by immunoblotting using anti-Pha13 or anti-ubiquitin antibody. (B) *P*. *aphrodite* was treated with H_2_O (Mock) or SA, and immediately followed by infiltration of DMSO (-) or MG132 (+). Total proteins were extracted from leaves of the treated samples at 3 h post-treatment, and were used for in vivo IP assay using anti-Pha13 antibodies. Samples after IP were analyzed by immunoblotting using anti-Pha13 antibody. (A and B) Extracted total proteins (input) stained by coomassie brilliant blue (CBB) served as a loading control.

Our data indicates that Pha13 confers self-E3 ligase activity and ubiquitin chain binding activity. To analyze whether SA stimulate the ubiquitination of Pha13 or binding to ubiquitinated protein, immunoprecipitation (IP) assay was performed to precipitate the Pha13 and ubiquitinated Pha13 in H_2_O (Mock) and SA treated *P*. *aphrodite* using anti-Pha13 antibody. Then, we used anti-Pha13 or anti-ubiquitin antibody for time course determination of Pha13 and ubiquitinated Pha13 (Fig 8A). We have repeated this experiments for more than 3 times with consistent results. Our data indicates that mock and SA treatment increased accumulation of the Pha13. Compared to mock-treated *P*. *aphrodite*, decreased accumulation of Pha13 was observed in SA-treated *P*. *aphrodite* at each time point (Fig 8A). When we used MG132 to inhibit 26S proteasome activity, the protein levels of Pha13 increased in SA treated- but not in mock treated-*P*. *aphrodite* (Fig 8B).

The results indicate that both mock and SA treatment increased the accumulation of Pha13; however, SA treatment may induce the degradation of Pha13 through 26S proteasome activity.

In addition, in our assay using anti-ubiquitin antibody to detect the precipitated protein(s), we detected a protein band with molecular weight of around 43 kD increased with time in both SA treated-*P*. *aphrodite* and mock treated-*P*. *aphrodite*. However, increased signals of the 43 kD protein band was detected more in SA treated- than in mock treated-*P*. *aphrodite* (Fig 8A). The results indicate that SA treatment can have multiple effects on Pha13 at protein levels.

### Subcellular localization of Pha13

Analysis of Pha13 amino acid sequence by the use of PredictProtein (https://www.predictprotein.org/) (Fig 1B) revealed putative nuclear localization signal. We therefore wondered whether Pha13 localized to the nucleus. To observe the subcellular localization of Pha13, we transiently expressed Pha13 fused to GFP either at the N- or C terminal, designated 35S::G-Pha13 and 35S::Pha13-G, respectively, in protoplasts isolated from *P*. *aphrodite*. Protoplasts were collected 24 h post-transfection and examined by confocal microscope. In about 50% of cells expressing G-Pha13 and Pha13-G, green fluorescence was observed exclusively in the nucleus (S5 Fig). Nucleus-specific green fluorescence was not observed in the GFP control vector.

### Overexpression of Pha13 in *Arabidopsis* enhances resistance to viral and bacterial pathogens

**Fig 9:**
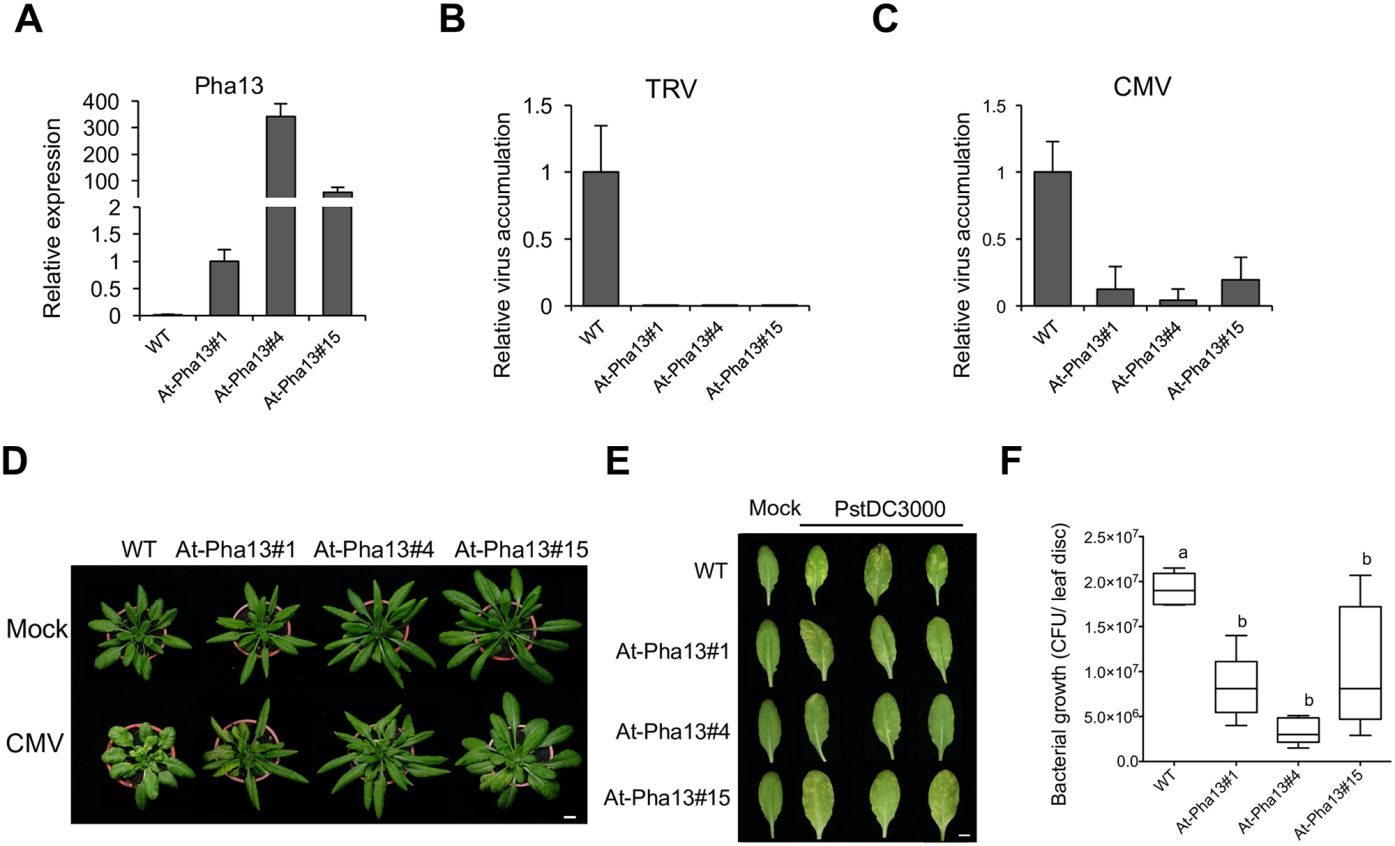
Overexpression of Pha13 in engineered transgenic *Arabidopsis* enhances resistance against viruses and bacteria. (A to C) Expression level of *Pha13* (A) and accumulation level of *Tobacco rattle virus* (TRV) (B) and *Cucumber mosaic virus* (CMV) (C) were analyzed by qRT-PCR from leaves of WT (Col-0) or transgenic *Arabidopsis*. Data represent mean ± SD; n = 4 biological replicates; *, *P* < 0.05, Student’s t-test compared to vector. *Actin* was used as an internal control for normalization. (D) Disease symptoms of WT (Col-0) or transgenic *Arabidopsis* inoculated with CMV at 9 dpi. Images are one representative plant from four replicates; Scale bar, 1 cm. (E) Disease symptoms of WT (Col-0) or transgenic *Arabidopsis* inoculated with 1 × 10^7^ cfu/ml *Pseudomonas syringae* pv. *tomato* DC30000 (PstDC3000) at 3 dpi. Images are three representative leaves from five plants; Scale bar, 1 cm. (F) The growth analysis of PstDC3000 in the infected leaves of WT or transgenic *Arabidopsis*. Data represent mean ± SD; n = 5 biological replicates. Different letters indicate statistically significant differences analyzed by one-way analysis of variance (ANOVA) Tukey’s test (*P* < 0.05).

Previously, phylogenetic analysis revealed that SAP proteins are evolutionarily conserved among plants [28]. To analyze whether Pha13 mediated plant immunity is conserved in plants, we transformed 35S promoter driven overexpression Pha13 (pPha13-oe) constructs into *Arabidopsis* (Col-0). Homozygous T3 plants derived from 3 T1 transgenic lines were selected for further disease resistance analysis. The overexpression of Pha13 was confirmed by qRT-PCR on the homozygote progenies (Fig 9A). All Pha13 overexpression transgenic *Arabidopsis* displayed higher shoot fresh weight and longer radius of leaf than *Arabidopsis* wild-type (S6A–S6C Fig) and confer an early-flowering phenotype (S6D and S6E Fig).

We inoculated *Tobacco rattle virus* (TRV), which does not cause symptoms on Col-0, by agroinfiltration to four wild-type and four Pha13 overexpressing *Arabidopsis*. The results revealed that TRV accumulation is dramatically decreased in all three Pha13 overexpressing transgenic lines at 9 days post-inoculation (dpi) (Fig 9B). In addition, we also mechanically inoculated *Cucumber mosaic virus* (CMV) to wild-type and Pha13 overexpressing *Arabidopsis*. The results showed that Pha13 overexpressing *Arabidopsis* greatly enhanced resistance to CMV (Fig 9C and 9D). In addition to viruses, we also inoculated *Pseudomonas syringae* pv. *tomato* DC30000 (PstDC3000) to five T3 progenies of At-pha13#1, At-pha13#4 and At-pha13#15. The three *Arabidopsis* Pha13 transgenic lines showed enhanced resistance to PstDC3000 compared to *Arabidopsis* wild-type at 3 dpi (Fig 9E and 9F).

### *AtSAP5*—An *Arabidopsis* homolog of Pha13 is involved in viral resistance

**Fig 10:**
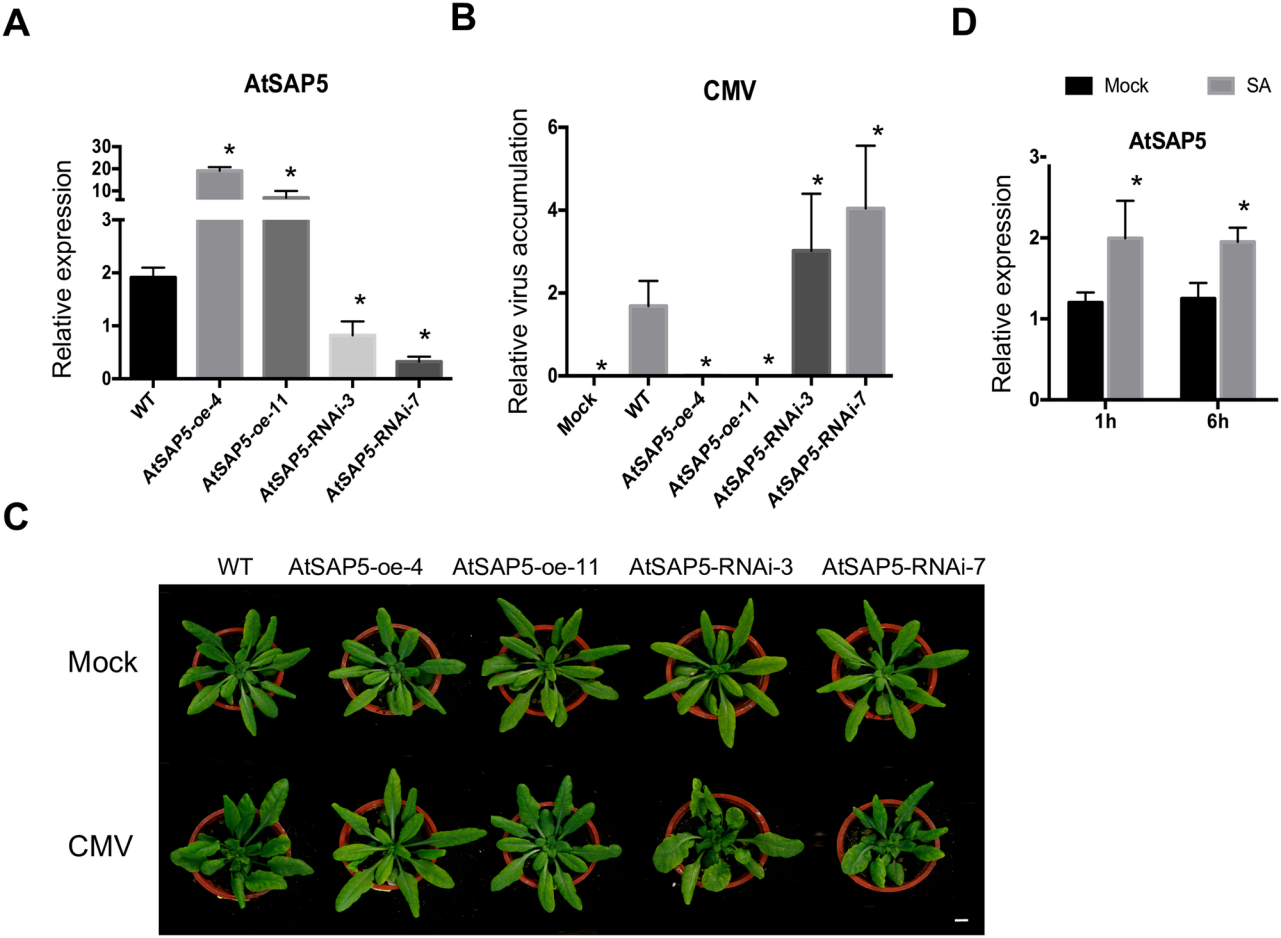
The *Arabidopsis* homologue of Pha13, *AtSAP5*, is induced by SA and involved in virus resistance. (A) The expression of *AtSAP5* were analyzed by qRT-PCR in the WT, AtSAP5 overexpression lines (AtSAP5-oe-4 and AtSAP5-oe-11), and RNAi lines (AtSAP5-RNAi-3 and AtSAP5-RNAi-7). Data represent mean ± SD; n = 3 biological replicates *, p<0.05, Student’s t-test compared to WT (B) The accumulation levels of *Cucumber mosaic virus* (CMV) were analyzed by qRT-PCR from leaves of WT inoculated with buffer (Mock), CMV-inoculated WT, or CMV-inoculated transgenic *Arabidopsis*. Data represent mean ± SD; n = 5 biological replicates *, p<0.05, Student’s t-test compared to WT (C) Disease symptoms of CMV inoculated WT or transgenic *Arabidopsis*. The experiments were repeated twice with similar results, and 5 plants were used in each inoculation. Photo of one representative plant from each inoculation taken at 9 days post inoculation are shown here; Scale bar, 1 cm. (D) Time-course expression of *AtSAP5* in SA-treated wild-type (WT, Col-0) *Arabidopsis*. Expression level of *AtSAP5* was analyzed by qRT-PCR from leaves at 1 and 6 h post-treatment. Water treatment was used as a mock control. Data represent mean ± SD; n = 3 biological replicates *, p<0.05, Student’s t-test compared to mock. *Actin* was used as an internal control for normalization.

Our phylogenetic analysis revealed that Pha13 is most related to *Arabidopsis AtSAP5* (S7 Fig). Therefore, we generated transgenic *Arabidopsis* (Col-0) to overexpress *AtSAP5*, and also generated RNAi lines to express hairpin RNA of *AtSAP5*. The over- and down-expression of *AtSAP5* were confirmed by qRT-PCR in two randomly-selected overexpression (AtSAP5-oe-4 and AtSAP-oe-11) and RNAi (AtSAP5-RNAi-3 and AtSAP5-RNAi-7) lines (Fig 10A). No obvious difference in phenotype was observed among the transgenic plants and WT (S8 Fig).

We mechanically inoculated CMV to WT (Col-0), AtSAP5-oe-4, AtSAP5-oe-11, AtSAP5-RNAi-3, and AtSAP5-RNAi-7. Total RNA was extracted from CMV inoculated-WT, -AtSAP5-oe-4, -AtSAP5-oe-11, -AtSAP5-RNAi-3 and -AtSAP5-RNAi-7, and used for qRT-PCR in the quantification of CMV. The results showed that CMV was detected in WT (Fig 10B). In comparison, the level of CMV was below our accurate detection limit in AtSAP5-oe-4 and AtSAP5-oe-11, whereas higher CMV accumulation was observed in AtSAP5-RNAi-3 and AtSAP5-RNAi-7 compared to WT (Fig 10B). While similar disease symptom was observed on CMV infected-WT, -AtSAP5-RNAi-3, and -AtSAP5-RNAi-7, no disease symptom on CMV-infected AtSAP5-oe-4 and AtSAP5-oe-11 were observed (Fig 10C). Our data indicates that AtSAP5 in *Arabidopsis* is also involved in virus resistance (Fig 10B and 10C).

### AtSAP5 is involved in the expression of NPR1 and NPR1-independent genes

**Fig 11:**
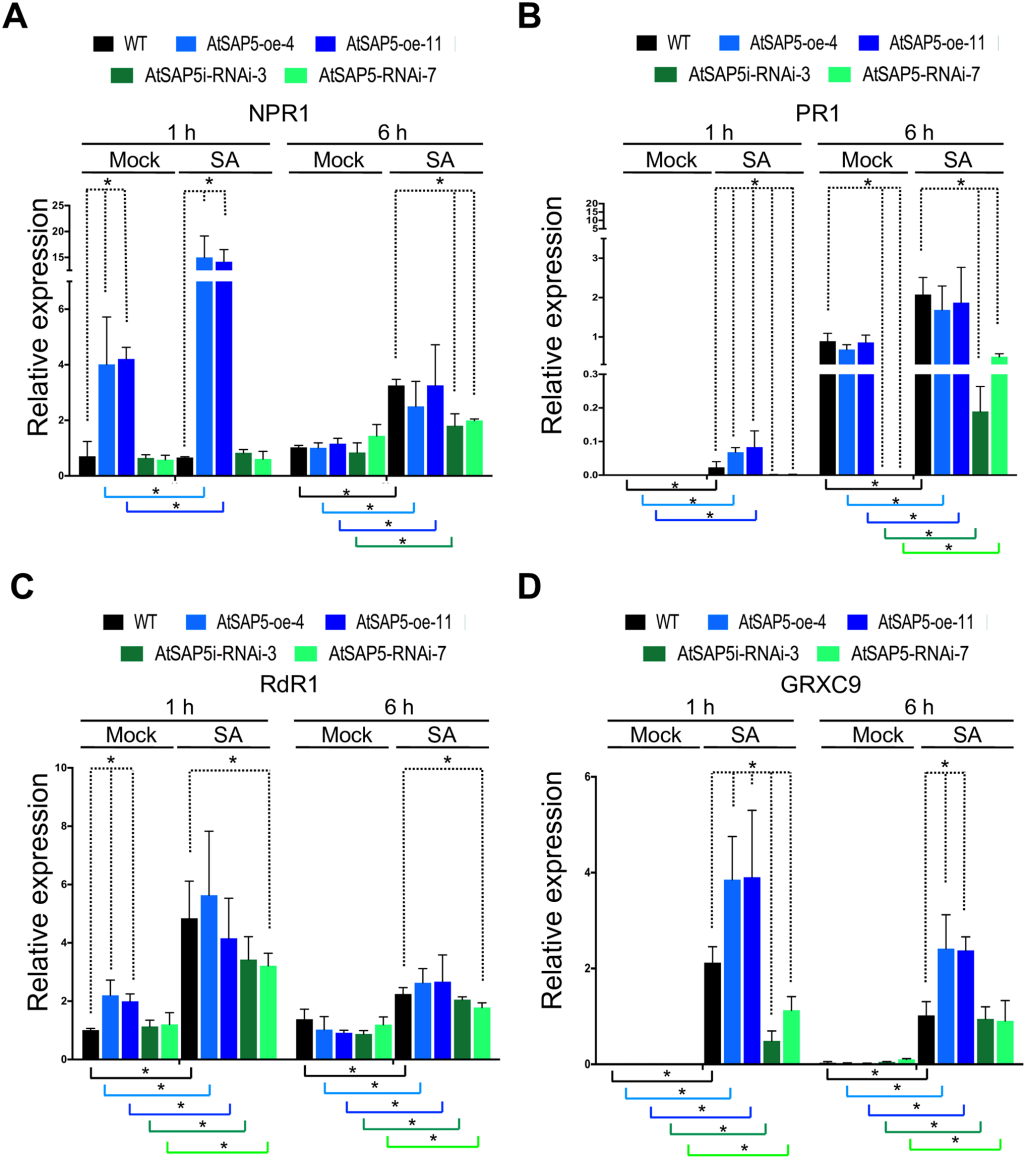
AtSAP5 is involved in the expression of NPR1 and NPR1-independent genes. (A-D) The expression level of *NPR1* (A), *PR1* (B), *RdR1* (C), and *GRXC9* (D) were analyzed by qRT-PCR in H_2_O (Mock) or SA-treated WT, AtSAP5 overexpression (AtSAP5-oe-4 and AtSAP5-oe-11) and RNAi lines (AtSAP5-RNAi-3 and AtSAP5-RNAi-7). Data represent mean ± SD; n = 3 biological replicates; *, p<0.05, Student’s t-test. The dashed lines indicate the sample with significant difference compared to WT. The colored lines indicate the significant difference was analyzed between corresponding samples treated with SA or mock. *Actin* was used as an internal control for normalization.

To analyze whether AtSAP5 also plays similar role as Pha13, total RNA was extracted from WT, AtSAP5-oe-4, AtSAP5-oe-11, AtSAP5-RNAi-3 and AtSAP5-RNAi-7, with H_2_O (Mock) or SA treatment and used for the detection of *AtSAP5*, *NPR1*, *PR1*, *RdR1* and *GRXC9* expression by qRT-PCR (Fig 11).

Our analysis also revealed that SA treatment induced the expression of *AtSAP5* at 1 and 6 hours post treatment (hpt) in WT (Fig 10D). The expression level of *NPR1* and *RdR1* is similar in WT, AtSAP5-oe-4, AtSAP5-oe-11, AtSAP5-RNAi-3 and AtSAP5-RNAi-7 without treatment (S9 Fig). The expression of *PR1* and *GRXC9* is below our accurate detection limit in WT, AtSAP5-oe-4, AtSAP5-oe-11, AtSAP5-RNAi-3 and AtSAP5-RNAi-7 without any treatment (S9 Fig).

With mock (H_2_O) treatment, higher expression of *NPR1* and *RdR1* was observed in AtSAP5-oe-4 and AtSAP-oe-11 (Fig 11A and 11C) as compared to H_2_O treated-WT at 1 hpt. With SA treatment, significantly higher expression of *NPR1* (1 hpt), *PR1* (1 hpt), and *GRXC9* (1 and 6 hpt) was observed in AtSAP5-oe-4 and AtSAP-oe-11 as compared to SA treated-WT (Fig 11A, 11B and 11D). No decreased expression of *NPR1*, *PR1*, *RdR1* and *GRXC9* was observed in AtSAP5-oe-4 and AtSAP-oe-11 as compared to WT regardless of the treatment or time-point (Fig 11A–11D).

With mock (H_2_O) treatment, decreased expression of *PR1* was observed in both AtSAP5-RNAi-3 and AtSAP5-RNAi-7 as compared to H_2_O treated-WT at 6 hpt (Fig 11B). With SA treatment, decreased expression of *NPR1* (6 hpt), *PR1* (1 and 6 hpt) and *GRXC9* (1 hpt) was observed in both AtSAP5-RNAi-3 and AtSAP5-RNAi-7, and decreased *RdR1* (1 and 6 hpt) expression was observed in AtSAP5-RNAi-7 as compared to SA treated-WT (Fig 11A–11D). No increased expression of *NPR1*, *PR1*, *RdR1* and *GRXC9* were observed in AtSAP5-RNAi-3 and AtSAP5-RNAi-7 as compared to WT regardless of the treatment or time point (Fig 11A–11D).

Collectively, our data using transgenic *Arabidopsis* overexpressing or silencing *AtSAP5* suggests that AtSAP5 is involved in the expression of NPR1 and NPR1-independent genes.

## Discussion

In this report, we provided evidence indicating that an orchid SAP gene, *Pha13*, serves pivotal roles in resistance to viruses through important but previously unidentified SA responsive transcriptional reprogramming of immune responsive gene(s). First, our analysis revealed the striking similarities between *Arabidopsis* and orchids in plant immune responses. Counterparts of the SA-dependent plant immune responsive genes found in *Arabidopsis* including *PR1*, *NPR1*, *RdR1*, and *GRX* were also identified in orchids (i.e. *PhaPR1*, *PhaNPR1*, *PhaRdR1*, and *PhaGRX*). Indeed, SA induces these orchid genes as they do in *Arabidopsis* counterparts (Figs 2D and 4A). The dependency on *PhaNPR1* for expression of *PhaPR1*, *PhaRdR1*, and *PhaGRX* are also similarly reported in *Arabidopsis* (Fig 4C). Taking these results together we can see that this central immunity is conserved across plants. Furthermore, we also found that overexpression of Pha13 in engineered transgenic *Arabidopsis* conferred resistance to various viruses and bacteria (Fig 9). Similarly, a previous report indicated that overexpression of a rice SAP gene, *OsSAP1*, in tobacco can enhance protection of plants against bacterial pathogen infection [33]. More importantly, we also demonstrated that *Arabidopsis* homolog of Pha13, AtSAP5, also play similar role in virus resistance and immune regulation (Figs 10 and 11). These findings together suggest that the downstream immune responsive pathways of SAPs are conserved among plants.

Previous reports have suggested that NPR1 responds to SA signal and is regulated mainly at the post-translational level [10, 16]. This mode of regulation allows plants to quickly respond to invading pathogens without undergoing transcription, which is particularly important in initial defense. Our study showed that RNA expression of *Pha13* is induced earlier than *PhaNPR1* during SA induction (Fig 2D), and both silencing and overexpression of Pha13 affected the RNA accumulation of *PhaNPR1* (Fig 2A and 2B), whereas silencing *PhaNPR1* did not affect the expression of Pha13 RNA in orchid (Fig 2C). These results support the notion that Pha13 plays a role in immune regulation downstream of SA and upstream of *PhaNPR1* (Fig 5C).

Interestingly, our data also indicated that in addition to participating in the expression of *PhaNPR1*, Pha13 is also involved in the expression of PhaNPR1-independent immune defense genes (Fig 5C). Overexpression of Pha13 in orchids affects at least 10639 genes (S1A Fig) including PhaNPR1 -dependent and–independent genes (S1 Table), which suggest a broad spectrum of regulation by Pha13.

Although Pha13 is involved in SA responsive transcriptional reprogramming of immune responsive genes, our biochemical analysis suggests that Pha13 does not directly function as a transcriptional regulator; rather, Pha13 may regulate the transcription of immune responsive genes in an indirect manner. This is because our analysis indicates that Pha13 confers ubiquitin binding and E3 ligase activity, and both activities are not directly involved in transcriptional activity.

Our data also suggests that SA participates in the regulation of Pha13 at the protein level, as our data indicates that while both mock and SA treatment increase the accumulation of *Pha13* RNA and proteins (Figs 2D and 8A); only SA treatment can induce the degradation of Pha13 through 26S proteasome activity (Fig 8B). In addition, an ubiquitinated protein band with molecular weight around 43 kD was immunoprecipitated with anti-Pha13 antibody in both SA treated-*P*. *aphrodite* and mock treated-*P*. *aphrodite*, and increased signals of the 43 kD protein band was detected in SA treated-*P*. *aphrodite* as compared to mock treated-*P*. *aphrodite* (Fig 8A). Although the identity and function of the 43 kD protein has yet to be identified, our data suggested that SA also regulates Pha13 at the protein level.

Proteins containing A20 and/or AN1 zinc finger domains are conserved among various organisms. Different numbers of A20/AN1 protein (from 1 to 19) exist in different organisms including protists, fungi, animals, and plants [28, 34]. The most well-known protein in this class, A20, plays a pivotal role in negative regulating central immune transcription factor, NF-kB, in human. Human A20 binds to multiple signaling proteins (substrates) upstream of NF-kB to interfere with the function of the substrate or modulating the ubiquitination of different substrates to regulate NF-kB [35, 36]. In addition to human A20 (contains 7 A20 domain), AWP1 and ZN216 (contain single A20 and AN1 domain) are also reported to have redundant but distinct functions in regulating NF-kB [37, 38]. Genetic studies have provided strong evidence indicating that A20/AN1 proteins are involved in abiotic stress in plant and known as stress associated proteins (SAPs) [28]. Among currently known plant SAPs, two SAP genes, *Arabidopsis AtSAP9* and rice *OsSAP1*, may be involved in plant immunity [33, 39]. Overexpression of rice OsSAP1 in tobacco enhanced the plant resistance to bacterial pathogen infection [33]. Transgenic *Arabidopsis* overexpressing AtSAP9 decrease resistance to a non-host bacterial pathogen [39]. However, the role of OsSAP1 and AtSAP9 in plant immunity remains elusive.

Human A20 and Rabex-5 (guanine nucleotide exchange factor) contain A20 domain but without AN1 domain; while ZNF216 and AWP1 more resemble Pha13, AtSAP5, and AtSAP9, which contain both A20 and AN1 domains. In human A20 and Rabex-5, the A20 domain exhibits both E3 ligase and ubiquitin binding ability [32, 40]. However, only ubiquitin binding ability has been reported for ZNF216 and AWP1 [41, 42]. In plants, AtSAP9, AtSAP5, TsSAP5 from wheat (*Triticum aestivum*) and Pha13 confer E3 ligase and/or ubiquitin binding ability (Figs 6 and 7) [39, 43–45]. Ubiquitin binding activity has been mapped to A20 domain on human A20, Rabex-5, ZNF216, AWP1, AtSAP5 and Pha13 (Fig 7) [35, 40–43]. The domain responsible for E3 ligase and ubiquitin binding ability of AtSAP9 and domain responsible for E3 ligase of TsSAP5 remain to be resolved; however, it has been demonstrated that A20 domain of AtSAP5 and Pha13 confer both E3 ligase and ubiquitin binding ability (Figs 6 and 7) [43, 44].

Human A20 bind K63 and M1 polyubiquitin chain through its fourth and seventh A20 domain. In plants, single A20 domain can bind to various polyubiquitins. AtSAP5 binds to M1, K48, and K63 polyubiqutin, with preference for K63 polyubiquitin; while AtSAP9 binds K48 and K63 polyubiquitin chain, and also with preference for K63 polyubiquitin [39, 43]. In comparison to human A20, AtSAP5, and AtSAP9, Pha13 has profound polyubiquitin binding ability. Our results revealed that Pha13 binds to M1, K6, K11, k29, k33, K48 and K63 polyubiquitin chain, and prefers binding to M1, K29 and K63 polyubiquitin (Fig 7B). The ability for Pha13 to bind diverse polyubiquitin chains suggests that Pha13 may bind to a greater number of polyubiquitinated proteins.

Our data indicated that Pha13 and human A20 share similar and distinct biochemical characteristics. Both Pha13 and human A20 exhibit E3 ligase and polyubiquitin chain binding activity. Human A20 binds to at least 10 substrates to regulate immunity through direct binding to proteins or ubiquitin chains on the proteins in regulating their ubiquitination status [46]. The profound binding activity of Pha13 to numerous different ubiquitin chains suggest that Pha13 may bind to multiple substrates in an even more elaborate regulation of plant resistance.

In comparison to A20 domain, less is known about the biological and biochemical function of AN1 domain in either animals or plants. Notably, strong E3 ligase activity have been reported on AN1 domain of AtSAP5 [44]; however, our analysis indicated that most E3 ligase activity is conferred on the A20 but not on AN1 domain of the Pha13 (Fig 6). The result shows that A20 and AN1 domains may confer different functions in various SAPs.

Collectively, we propose a model that, in the initial SA response, post-translational modification of PhaNPR1 quickly turns on immune response gene(s) (PhaNPR1-dependent) such as *PhaPR1* (Fig 2D). In addition, SA regulate *Pha13* at both transcriptional and post-translational levels, and leads to broad transcriptional reprograming of immune responsive genes including PhaNPR1-dependent and PhaNPR1-independent genes. Our data suggests that SA mediates both NPR1 post-translational and Pha13-mediated plant immune response in a temporally controlled and functionally cooperative manner (Fig 5C). Recently, a member of the CCCH zinc finger domain family, oxidation related zinc finger 1, was reported to play a positive role in the SA dependent, NPR1-independent defense response against bacterial pathogen in *Arabidopsis* [47]. Although the biochemical properties of oxidation related zinc finger 1 remain elusive, the findings suggest that different zinc finger domain containing proteins may play an important role in SA mediated NPR1-indepdent immune pathway.

In our study, we identified an ancient conserved immune regulator, Pha13, in orchids that is crucial for SA–governed defense. SA regulates Pha13 at both transcriptional and post-translational levels. Moreover, transgenic *Arabidopsis* overexpressing orchid Pha13 also confers greater resistance to different pathogens (Fig 9), suggesting Pha13 regulated the downstream immune response pathway(s) that is conserved in both monocots and dicots. We also demonstrated that *Arabidopsis* homolog of Pha13, AtSAP5, also plays similar role in virus resistance and immune regulation (Figs 10 and 11). Our findings greatly enhanced the understanding in the regulation of the SA-mediated immune responses among plants, providing important information in the development of plant resistance to a broad-spectrum of pathogens.

## Material and methods

### Subject materials and growth conditions

The commercial orchid variety, *Phalaenopsis aphrodite var*. *Formosa*, was purchased from Taiwan Sugar Research Institute (Tainan, Taiwan). *P*. *aphrodite*, *P*. *equestris* and transgenic *P*. *equestris* (35S∷FLAG-Pha13) plants were all first tested for two prevalent orchid viruses, *Odontoglossum ringspot virus* (ORSV) and CymMV, as detected by RT-PCR with primer pairs, ORSV-F/ORSV-R and CymMV-F/CymMV-R (S3 Table), before maintaining in a greenhouse with a controlled 12-h photoperiod (200 μmol m^-2^s^-2^) at 25°C/25°C (day/night). The *Arabidopsis* WT (Col-0) and all transgenic *Arabidopsis* were maintained in a greenhouse with a controlled 12-h photoperiod (200 μmol m^-2^s^-2^) at 22°C/22°C (day/night) for ~four weeks before analysis. *Cucumber mosaic virus* isolate 20 was maintained in the *Arabidopsis thaliana* (Col-0) as inoculation source in our study. The infectious clones of *Cymbidium mosaic virus* (pCambia-CymMV) and *Tobacco rattle virus* (pTRV1 and pTRV2) were transformed into *Agrobacterium tumefaciens* C58C1 (pTiB6S3ΔT)^H^ (described below) for inoculation of Orchid or *Arabidopsis* through agroinfiltration. *E*. *coli* strains BL21 was grown on Luria broth (LB) agar plates or in LB broth. *Pseudomonas syringae* pv. *tomato* DC30000 was grown in the King’s B medium (20 g/L proteose peptone, 1.5 g/L K_2_HPO4, 10 ml glycerol, and 1.5 g/L MgSO4 •7H_2_O, pH 7.0). *Saccharomyces cerevisiae* strain AH109 was grown on the YPAD (Yeast extract 10 g/L, Peptone 20 g/L, Dextrose 20 g/L, and Adenine sulfate 0.4 g/L) agar plate or YPAD broth.

### Phytohormone treatment

Sodium salicylate (50 mM) (Sigma), methyl jasmonate (45 μM) (Sigma), and aminocyclopropanecarboxylic acid (660 μM) (Sigma) were directly rubbed on leaves of *P*. *aphrodite* by cotton swab. Leaf samples were collected at 0 h, 1 h, 3 h, 6 h, 12 h, 24 h, 48 h and 72 h after treatment. For *Arabidopsis*, 1 mM SA was sprayed on the leaves, and samples were collected at 1 h, 6 h, and 24 h after treatment.

### Sequence analysis

The protein domains of Pha13 were analyzed by use of PROSITE database of ExPASy Proteomics Server (http://ca.expasy.org/) and Conserved Domain Database of NCBI database (http://www.ncbi.nlm.nih.gov/). The predicated nuclear localization signal of Pha13 was analyzed by use of PredictProtein (https://www.predictprotein.org/)

### Phylogenetic tree analysis

Phylogenetic analysis of Pha13 was conducted with 12 orchid A20/AN1 domain containing stress associated proteins (SAPs) and previously characterized 14 SAPs from *Arabidopsis thaliana* and 18 SAPs from *Oryza sativa*. The sequence alignment was performed by use of the Clustal W algorithm of the DNASTAR MegAlign software (DNASTAR, WI, USA). An unrooted phylogenetic tree was constructed using the neighbor-joining method by MEGA5 with 1000 bootstrap replicates. The sequences of SAPs from orchids, *A*. *thaliana*, and *O*. *sativa* were obtained from the websites, Orchidstra database (http://orchidstra2.abrc.sinica.edu.tw/), TAIR (http://www.arabidopsis.org) and the Rice Genome Annotation Project (http://rice.plantbiology.msu.edu), respectively. The accession of each gene used for analysis is indicated in S7 Fig.

### RNA isolation, semi-quantitative and qRT-PCR analysis

Total RNA was extracted as described previously [27]. For semi-quantitative RT-PCR, 1 μg of total RNA and oligo (dT) primer were used to synthesize the cDNA. The PCR was performed using the gene-specific primer pairs (S3 Table). The results of semi-quantitative RT-PCR were analyzed by ImageJ software for the relative quantification. For qRT-PCR, cDNA was synthesized from 500 ng of DNA-free RNA and oligo (dT) by use of PrimeScript RT Reagent Kit (Perfect Real Time) (Takara Bio) following the manufacturer’s instructions (Takara Bio). The cDNA template was used for qPCR by use of SYBR Premix EX Taq II (Ti RNase H Plus) Kit (Takara Bio) in an ABI Prism 7500 sequence detection system (Applied Biosystems). The *PhaUbiquitin 10* or *Actin* was used as an internal quantification control. The primer pairs used in this study are listed in (S3 Table).

### Construction of transient silencing and virus-induced gene-silencing vectors

For construction of transient silencing vector of Pha13, the oligonucleotide pair Pha13-hpRNA-F/Pha13-hpRNA-R (S3 Table) was used to generate the hairpin dsDNA fragments. The hairpin dsDNA fragments were cloned into the Gateway entry vector pENTR/D-TOPO (Thermo Fisher-Scientific) following the manufacturer’s instructions to generate pENTR-Pha13-hpRNA. Then, LR Gateway cloning reaction (Thermo Fisher-Scientific) was conducted to transfer the hairpin RNA fragments from pENTR-Pha13-hpRNA into 35S promoter driven pB7GWIWG2(I) [48] to obtain phpPha13. The method for construction of transient silencing vector of *PhaNPR1*, *PhaRdR1*, and *PhaGRX* was similar to that described above, except the oligonucleotide pairs PhaNPR1-hpRNA-F/PhaNPR1-hpRNA-R, PhaRdR1 -hpRNA-F/PhaRdR1-hpRNA-R, and PhaGRX-hpRNA-F/ PhaGRX-hpRNA-R (S3 Table) were used to generate the hairpin dsDNA fragments.

For construction of virus-induced gene-silencing vector, plant RNA was used as a template to amplify the fragments of *Pha13* by RT-PCR with the primer pair attB1-Pha13-F/attB2-Pha13-R (S3 Table). The amplified fragments were cloned into the pCambia-CymMV-Gateway vector [27] by the BP Clonase II enzyme mix (Thermo Fisher-Scientific) to generate pCambia-CymMV-Pha13.

### Construction of Pha13 overexpression vector

For construction of Pha13 transient overexpression vector, plant total RNA was used as a template to amplify the N terminal FLAG tagged of Pha13 by RT-PCR with the primer pairs FLAG-Pha13ORF-F/Pha13ORF-R (S3 Table). The FLAG-Pha13 fragments were cloned into the Gateway entry vector pENTR/D-TOPO (Thermo Fisher-Scientific) following the manufacturer’s recommendations to generate pENTR-FLAG-Pha13. Then, LR Gateway cloning reaction (Thermo Fisher-Scientific) was used to transfer the FLAG-Pha13 fragments from pENTR-FLAG-Pha13 into the 35S promoter driven overexpression vector, pK2GW7 [48], to obtain pPha13-oe. For generation of A20 and/or AN1 mutant on pPha13-oe (Fig 1C and 1D), site-directed mutagenesis was conducted by QuikChange Site-Directed Mutagenesis Kit (Agilent Technologies). For A20 mutant, we substituted the conserved 3^rd^ and 4^th^ cysteine to glycine at A20 (C29G and C32G). For AN1 mutant, we substituted the conserved 3^rd^ cysteine and 1^st^ histidine to glycine at AN1 (C111G and H121G). The A20 mutated clone and AN1 mutated clone was designated pPha13A20m and pPha13AN1m, respectively. The A20 and AN1 double mutated clones was designated pPha13A20mAN1m. Primer pairs used for site directed mutagenesis are listed in S3 Table.

### Transgenic *Phalaenopsis* orchid and *Arabidopsis* plants

For construction of overexpression vector to generate transgenic *Phalaenopsis* orchid, the FLAG-Pha13 fragment was transferred from pENTR-FLAG-Pha13 (described above) into binary vector, pH2GW7 [48], to obtain pHPha13. pHPha13 was used to generate transgenic *P*. *equestris* orchid using the method described by Hsing *et al*. [49]. For the construction of overexpression and RNAi vector of AtSAP5, pAtSAP5-oe or phpAtSAP5-RNAi, we followed similar method as described above for the construction of pPha13-oe and phpPha13, except the primers used to amplify the full length and the fragment of AtSAP5 were AtSAP5ORF-F/AtSAP5ORF-HA-R (for overexpression vector, pAtSAP5-oe) and AtSAP5-RNAi-F/AtSAP5-RNAi-R (for RNAi vector, phpAtSAP5-RNAi; S3 Table).

For the generation of transgenic *Arabidopsis*, the plants were transformed using the floral dip method with *Agrobacterium tumefaciens* strain GV3101 carrying the pPha13-oe, pAtSAP5-oe, or phpAtSAP5-RNAi to generate the transgenic plants.

### Agroinfiltration

Agroinfiltration on orchid was conducted as previously described [27], with modification. pCambia-CymMV, pB7GWIWG2, pK2GW7 and their derivatives and were transformed into *Agrobacterium tumefaciens* C58C1 (pTiB6S3ΔT)^H^ by electroporation. Briefly, *A*. *tumefaciens* strains were incubated at 28°C until reaching an OD_600_ to 1.0. After centrifugation, 20 ml AB-MES medium (17.2 mM K_2_HPO_4_, 8.3 mM NaH_2_PO_4_, 18.7 mM NH_4_Cl, 2 mM KCl, 1.25 mM MgSO_4_, 100 μM CaCl_2_, 10 μM FeSO4, 50 mM MES, 2% glucose (w/v), pH 5.5) with 200 μm acetosyringone [50] was used to re-suspend the cells. After culturing overnight, 2 ml of infiltration medium containing 50% MS medium (1/2 MS salt supplemented with 0.5% sucrose (w/v), pH 5.5), 50% AB-MES and 200 μm acetosyringone [50] were used for infiltration.

For the agroinfiltration on *Arabidopsis*, same method was used as described above except pTRV1 and pTRV2 were individually transformed into *A*. *tumefaciens* C58C1 (pTiB6S3ΔT)^H^ by electroporation, and overnight culture of *A*. *tumefaciens* containing pTRV1 and pTRV2 was adjusted to OD_600_ of 0.5 and mixed at 1:1 ratio prior to infiltration.

### *Cymbidium mosaic virus* (CymMV), *Cucumber mosaic virus* (CMV), and *Tobacco rattle virus* (TRV) inoculation and accumulation assay

To assay the effect of *Pha13*, *PhaRdR1*, or *PhaGRX* in CymMV accumulation, we first inoculated CymMV in *P*. *aphrodite*. For inoculation of CymMV, agroinfiltration (described above) was performed to infiltrate *A*. *tumefaciens* carrying pCambia-CymMV in the leaf tip of *P*. *aphrodite*. The CymMV-infected *P*. *aphrodite* were maintained at least 14 days before further analysis. To assay the effect of transient silencing (*Pha13*, *PhaRdR1*, or *PhaGRX*) or transient overexpression (*Pha13*, or their derived mutants), *A*. *tumefaciens* carrying the control vector pB7GWIWG2 (for silencing), pK2GW7 (for overexpression), silencing vectors or overexpression vectors (described above) were infiltrated into the leaves. After agroinfiltration, a pair of disks (6 mm diameter) were immediately (defined as 0 dpi) collected from both the control and assay vector infiltrated regions. After 5 dpi, another pair of disks were collected from the same infiltrated region. Total RNA extracted from the samples was used as a template to analyze the accumulation of CymMV by use of qRT-PCR. The ratio of CymMV accumulation at 0 dpi to 5 dpi was calculated for relative quantification.

For TRV inoculation, agroinfiltration (as described above) was performed by infiltrating *A*. *tumefaciens* carrying pTRV1 or pTRV2 (1:1) to three leaves of four-week-old *Arabidopsis* by use of syringe. After 9 dpi, total three disks from three different distal leave were collected and quantified the accumulation of TRV by use of qRT-PCR.

For inoculation with CMV, CMV-infected *Arabidopsis* leaves were ground with 0.01 M potassium phosphate buffer by mortar and pestle for use as the inoculation source. Four-week-old *Arabidopsis* leaves were inoculated mechanically (pre-dusted with 300-mesh Carborundum) with the CMV inoculation source. After 9 dpi, three disks from three different distal leave were collected and CMV accumulation was analyzed by use of qRT-PCR.

### Microarray analysis

Total RNA was extracted from leaves of *P*. *aphrodite* infiltrated with agrobacterium carrying vector (pK2GW7) or pPha13-oe 5 days after infiltration. For microarray, 0.2 μg of total RNA was amplified by a Low Input Quick-Amp Labeling kit (Agilent Technologies) and labeled with Cy3 (CyDye, Agilent Technologies, USA) during the in vitro transcription process. An amount of 0.6 μg of Cy3-labled cRNA was fragmented at 60°C for 30 minutes. Corresponding fragmented labeled cRNA was then pooled and hybridized to Agilent *P*. *aphrodite* 8 × 60K Microarray (Agilent design ID: 033620) [51] at 65°C for 17 hours. After washing and drying steps, the microarrays were scanned with an Agilent microarray scanner (Agilent Technologies) at 535 nm for Cy3. The array image was analyzed by Feature Extraction software version 10.7.1.1 using the default setting. For the microarray analysis, data were analyzed from 3 biological repeats using GeneSpring (Agilent Technologies, http://www.agilent.com). Pha13-responsive differentially expressed genes (DEGs) were identified based on significance compared to vector control (unpaired t test *P* < 0.05). Microarray data was deposited in the public repository GEO database (https://www.ncbi.nlm.nih.gov/geo/) with accession number GSE93248.

### Gene ontology analysis

Gene ontology (GO) classification was conducted with all the Pha13 -responsive DEGs by use of GO enrichment analysis algorithm of gene ontology database (http://geneontology.org/). All the Pha13-responsive DEGs were categorized into subcategories of biological process GO terms.

### Isolation and detection of Pha13 and Pha13 A20 and/or AN1 domain mutant protein

Total proteins were extracted from leaves of transgenic orchid or leaves of orchids infiltrated with agrobacterium carrying vector (pk2GW7), overexpression clones of wild-type Pha13 (pPha13-oe), or the respective A20 and/or AN1 mutant clone (pPha13A20m, pPha13AN1m or pPha13A20mAN1m) as previously described [52] with some modification. The boiled extraction buffer (4 M urea, 5% SDS, 15% glycerol, 100 mM Tris-HCl, pH 8, with freshly added 2 mM phenylmethylsulfonyl fluoride, 2 mg mL-1 and 1X complete protease inhibitor [Roche]) was used to extract the total proteins. The Pha13 or Pha13 A20 and/or AN1 domain mutant protein was analyzed by immunoblotting with antibody against Pha13 followed by HRP conjugated anti-rabbit antibodies (Abcam). The protein level of tubulin (loading control) was analyzed by immunoblotting with antibody against tubulin followed by HRP conjugated goat anti-mouse antibodies (GE Healthcare Life Sciences).

### Construction, expression, and purification of recombinant proteins

Full-length Pha13, or mutant cDNA were amplified by PCR with primer pairs, *Nde*I-Pha13-F/*Not*I-Pha13-R (S3 Table), with previously described Pha13 overexpression or mutated clones as templates. PCR amplified gene fragments were cloned into the pET24b expression vector (Novagen) with fused C-terminal histidine tag (His-tag) to produce protein expression plasmids, pETPha13, pETPha13A20m, pETPha13AN1m and pETPha13A20mAN1m. The constructed plasmids were transformed into *Escherichia coli* strain BL21 (DE3) for protein expression. Bacteria were cultured at 37°C to an OD_600_ of 0.5 and transferred to 25°C for 1.5 hours for Pha13, Pha13A20m, Pha13AN1m, and Pha13A20mAN1m protein induction. Protein induction was performed by addition of isopropylthio-β-galactoside (IPTG; Sigma) to a final concentration of 1 mM. His-tagged recombinant protein was purified by TALON Superflow (GE Healthcare Life Sciences) according to the manufacturer’s description. The elution was carried out with 250 mM imidazole (Sigma).

### E3 ubiquitin ligase activity assay

In vitro ubiquitination assays were performed as described [33] with modification. An amount of 3 μg purified His-tagged recombinant proteins (described above) were used for each ubiquitination reaction. Reactions were incubated at 30°C for 3 hours and analyzed by SDS-PAGE followed by immunoblot analysis. Blots were probed using anti-FLAG antibodies (Sigma) followed by HRP conjugated goat anti-mouse antibodies (GE Healthcare Life Sciences).

### Ubiquitin-binding assay

Full-length AtSAP5 cDNA was amplified by PCR with primer pairs, NdeI-AtSAP5-F/XhoI-AtSAP5-R (S3 Table), and were cloned into pET24b following the same approach as the cloning of pETPha13 described above to generate the pETAtSAP5. His-tagged AtSAP5, Pha13 and Pha13A20m recombinant proteins were expressed by *E*. *coli* and purified with affinity resin. Purified recombinant proteins (60 μg) were immobilized on magnetic beads (60 μl) using Mag-beads Carboxyl Labeling kit (Toolsbiotech) following the manufacturer’s instructions. Recombinant proteins (6 μl) conjugated magnetic beads were incubated with 4 μg of linear, K6, K11, K29, K33, K63-inked tetraubiquitin chains or K48-linked polyubiquitin (Ub3-Ub7) chains (BostonBiochem) for 18 h at 4°C in pull-down buffer (20 mM Tris–HCl pH 7.5, 150 mM NaCl, 10% glycerol, 1% Triton X-100, 10 mM ZnSO_4_, 0.5 mM DTT). After pull-down, the beads were washed three times with pull-down buffer. The pull-down proteins were boiled with SDS-sample buffer and analyzed by SDS-PAGE and immunoblotting with anti-ubiquitin antibody (Research and Diagnostic Systems).

### Immunoprecipitation (IP)

The IP assay for Pha13 was performed using total proteins extracted from 0.25 g of *Phalaenopsis* orchid leaves with 200 ul immunoprecipitation buffer (50 mM Tris-pH 7.5, 0.1% NP40, 10 mM MgCl_2_, 150 mM NaCl, 10 uM MG132, 1X complete protease inhibitor [Roche]). The extract was centrifuged at 12000 ×*g* for 10 min at 4 °C. Then, the supernatants were transferred to the non-stick tube, and pre-cleared with protein A sepharose beads (GE Healthcare Life Sciences) 1 hours at 4 °C with gentle shaking. After, the extract was centrifuged at 1500 ×*g* for 2 min at 4 °C, the supernatant was further incubated with the anti-Pha13 antibodies for 2 hours at 4 °C with gentle shaking, followed by incubation with protein A sepharose beads (GE Healthcare Life Sciences) for 2 hours at 4 °C with gentle shaking. The beads were washed three times with ice-cold immunoprecipitation buffer and eluted by SDS-PAGE sampling buffer. The eluted proteins were analyzed by immunoblotting using the anti-Pha13 antibody and anti-ubiquitin antibody (Research and Diagnostic Systems).

### Protein degradation assays

For the degradation assay of Pha13, the leaves of *P*. *aphrodite* were treated with H_2_O (Mock) or SA, and immediately followed by infiltration of DMSO or 40 uM MG132. Total proteins were extracted from the treated samples at 3 hours post-treatment and followed by the IP assay using anti-Pha13 antibodies (described above). The eluted proteins were analyzed by immunoblotting using the anti-Pha13 antibody.

### Preparation and transfection of protoplasts

For construction of vectors used for subcellular localization analysis, the primer pairs, Pha13-ORF-F/Pha13-ORF-R and Pha13-ORF-F/Pha13-ORF-NONSTOP-R (S3 Table) were used to amplify 2 sets of Pha13 ORF (with or without stop codon). All the amplified ORF fragments of *Pha13* were cloned into the Gateway entry vector pCR 8/GW/TOPO Gateway (Thermo Fisher-Scientific) following the manufacturer’s recommendations to generate pCR8-Pha13 and pCR8-Pha13-NONSTOP. Then, LR Gateway cloning reaction (Thermo Fisher-Scientific) was used to transfer the ORF fragment of *Pha13* from pCR8-Pha13 into p2FGW7 driven by 35S promoter [48] to obtain N-terminal GFP fused clones (pG-Pha13). To obtain C-terminal GFP-fused clones (pPha13-G), we transferred pCR8-Pha13-NONSTOP into p2GWF7. Protoplast isolation and transfection were as described [27]. Transformed protoplasts were detected for florescence signals by confocal microscopy (Zeiss LSM 780, plus ELYRA S.1) with excitation at 488 nm and emission at 500 to 587 nm for GFP, and excitation at 543 nm and emission at 600 to 630 nm for mCherry.

### Yeast two-hybrid screening

Full-length Pha13 cDNA was amplified by PCR with primer pairs, *Nde*I-Pha13-F/*Eco*RI-Pha13-R (S3 Table). The fragments were cloned into the pGBK vector (Clontech) with fused N-terminal GAL4 DNA binding domain to produce pGBKPha13 plasmid as a bait vector. pGBKPha13 was transformed into AH109 yeast strain as competent cells for yeast two-hybrid cDNA library screening. Total RNA isolated from *Phalaenopsis* leaf tissue (24 hours after SA treatment) was used for cDNA library construction. The cDNA library was generated with a Make Your Own “Mate & Plate” Library System (Clontech) following the user manual. Candidate yeast colonies were picked up PCR amplification with primer pairs, T7promoter/3’ ADprimer (Clontech). Amplified DNA fragments were sequenced, and the sequences were used in a blast search on an orchid database (http://orchidstra2.abrc.sinica.edu.tw/) to identify corresponding genes. Full lengths of candidate genes were amplified with RT-PCR (S3 Table) and cloned into pGAD vector for further confirmation analysis. Yeast strains containing the appropriate bait and prey plasmids were cultured in liquid 2-dropout medium (leucine^-^ and tryptophan^-^) overnight. The overnight yeast culture was diluted to an OD_600_ of 0.06 and spotted on selection plates (containing histidine^-^, leucine^-^, tryptophan^-^ and 5-bromo-4-chloro-3-indolyl-alpha-D-galactopyranoside) for growth assay. Fragments of different N-terminus deletions of Pha13 were generated with restriction enzyme digestions of *FseI* (NEB), *ApaI* (NEB), *Sgr*AI (NEB), BtgI (NEB), or *PspXI* (NEB). Fragments of C-terminus deletions were generated by PCR amplification using primer pairs, Nd13Fs412F/*Eco*RI-Pha13-R, Nd13Ap361F/*Eco*RI-Pha13-R, Nd13Sg247F/ *Eco*RI-Pha13-R, Nd13Bt136F*/Eco*RI-Pha13-R, and Nd13Ps88F*/Eco*RI-Pha13-R (S3 Table). Pha13 A20 domain was amplified by PCR with primer pairs, Nd13A20F/BH13A20R (S3 Table). Pha13 AN1 domain was amplified with primer pairs, Nd13AN1F/BH13AN1R (S3 Table). The yeast two-hybrid assay of the truncated Pha13 fragments were conducted as above.

### *Pseudomonas syringae* pv. *tomato* DC30000 (PstDC3000) inoculation and quantification assay

Four-week-old *Arabidopsis* were dipped in liquid suspension of 10^7^ cfu/mL PstDC3000 in 10 mM MgSO4 containing 0.01% Silwet L-77 (Lehle Seeds) for 5 min. After inoculation, plants were kept at 100% relative humidity. For bacterial population quantification, three discs were collected from individual inoculated-plants after 3 days post-inoculation and grounded in sterile water. Serial dilution was performed and the King’s B medium containing 100 ug/ml rifampicin for colony counting.

### Quantification and statistical analysis

Data are presented as mean ± SD. The pair-wise t-test was performed to analyze the statistical significance between samples. The one-way analysis of variance (ANOVA) followed by Tukey’s test was performed to analyze the statistical significance for data of Pha13 expression level in different tissues and bacterial pathogen growth. In transient overexpression analysis, three plant replicates with expression level of at least 40% increase in Pha13 or the respective A20 and/or AN1 mutant, compared to vector control, were used for statistical analysis.

### Accession numbers

Pha13 (PATC148746), PhaPR1 (PATC126136), PhaNPR1 (PATC135791), PhaRdR1; (PATC143146), PhaGRX (PATC068819), PhaUBQ10 (PATC230548), PhaJAZ1 (PATC141437), PhaACO2 (PATC139319), AtActin (At3G18780), AtSAP5 (AT3G12630), OsSAP3 (LOC_Os01g56040.1), OsSAP5 (LOC_Os02g32840.1).

## Supporting information

S1 Table *Pha13*-responsive differentially expressed genes (DEGs) compared to vector control are classified into NPR1-dependent and -independent groups following previous studies. (PDF)

S2 Table Pha13 interacting proteins identified by yeast-two-hybrid screening. (PDF)

S3 Table Primers and probes used in this study. (PDF)

S1 File The sequence of Pha13 coding region. (PDF)

S1 Fig Transcriptional analysis of Pha13 transiently overexpressed leaves of *P*. *aphrodite*. (A) The number of up- or down-regulated differentially expressed genes (DEGs) in Pha13 transiently overexpressed leaves of *P*. *aphrodite*. (B) Gene Ontology (GO) analysis of up- and down-regulated DEGs in Pha13 transiently overexpressed leaves of *P*. *aphrodite*. The DEGs are classified into subcategories of biological process GO terms (X-axis). The Y-axis represents the number of genes.
(TIF)

S2 Fig Transient overexpression of Pha13 leads to the induction of *PhaNPR1*, *PhaRdR1*, and *PhaGRX*. Expression level of *Pha13*, *PhaNPR1 PhaRdR1*, and *PhaGRX* were analyzed by qRT-PCR from leaves of *P*. *aphrodite* infiltrated with agrobacterium carrying vector (Vector), or plasmid to overexpress Pha13 (Pha13-oe). The RNA level of vector was set to 1. Data represent mean ± SD; n = 3 biological replicates; *, *P* < 0.05, Student’s t-test compared to vector. *PhaUbiquitin 10* was used as an internal control for normalization.
(TIF)

S3 Fig The detection of Pha13 and A20 and/or AN1 mutant of Pha13. Leaves of *P*. *aphrodite* was infiltrated with agrobacterium carrying vector (pK2GW7), overexpression clones of wild-type Pha13 (pPha13-oe), or the respective A20 and/or AN1 mutant clone (pPha13A20m, pPha13AN1m or pPha13A20mAN1m). Total proteins extracted from the infiltrated leaves were used for immunoblotting analysis with the use of anti-Pha13 antibody (Anti-Pha13). The anti-tubulin antibody (Anti-Tub) was used as a loading control.
(TIF)

S4 Fig A summary of Pha13 functions in SA-induced plant immune response pathways. Pha13 E3 ligase activity and effects on expression of *PhaNPR1*, *PhaRdR1*, *PhaGRX*, and accumulation level of CymMV. Silencing and overexpression of wild-type Pha13 or derived mutant clones (Pha13A20m, Pha13AN1m, and Pha13A20mAN1m) in *P*. *aphrodite* are depicted. The oval circle and rectangle indicate the A20 and AN1 domain, respectively. The “X” symbol indicates the mutated A20 and/or AN1 domain(s). The strength of E3 ligase activity is indicated with “+” or “-”. Gene(s) -up and -down regulation is indicated with blue arrows pointing up and down, respectively. Unaffected gene expression is indicated with a horizontal blue line.
(TIF)

S5 Fig Subcellular localization of Pha13. Green fluorescent protein (GFP), N- and C- terminal GFP-fused Pha13 (G-Pha13 and Pha13-G) were transfected either alone or co-transfected with nucleus localization signal fused red fluorescence protein (NLS-RFP) in protoplasts of P. aphrodite. Fluorescence was detected by confocal microscopy after transfection. Scale bars represent 10 μm.
(TIF)

S6 Fig Developmental phenotypes of the homozygous T3 transgenic *Arabidopsis* (35S::FLAG-Pha13). (A to C) The developmental phenotypes of 25-day–old T3 progenies (35S∷FLAG-Pha13) derived from three T1 transgenic lines, At-Pha13#1, At-Pha13#4, and At-Pha13#15 were presented on A. Shoot fresh weight (B) and radius (C) of the plants were analyzed. (D and E) The total number of rosette leaves of 42-day-old T3 progenies (35S∷FLAG-Pha13) were measured (D) and photos of representative plants are shown on (E). A-E. On A, scale bar, 1 cm. For B and C, data represent mean ± SD; n = 7 biological replicates; *, *P* < 0.05, Student’s t-test compared to WT. For D, data represent mean ± SD; n = 10 biological replicates; *, *P* < 0.05, Student’s t-test compared to WT. On E, scale bar, 5 cm.
(TIF)

S7 Fig Phylogenic analysis of A20/AN1 proteins from *Phalaenopsis aphrodite*, *Arabidopsis thaliana* and *Oryza sativa*. The unrooted phylogenetic tree was constructed using the Clustal X program and neighbor-joining method by MEGA5 with 1000 bootstrap replicates. A20/AN1 zing finger proteins derived from *P*. *aphrodite*, *A*. *thaliana*, and *O*. *sativa* are indicated with the accession number. The branch where Pha13 is located is indicated with a grey hexagonal box. The sequences of SAPs from orchids, *A*. *thaliana* and *O*. *sativa* were obtained from the websites, Orchidstra database (http://orchidstra2.abrc.sinica.edu.tw/), TAIR (http://www.arabidopsis.org), and the Rice Genome Annotation Project (http://rice.plantbiology.msu.edu), respectively. The accession of each genes is indicated.
(TIF)

S8 Fig Developmental phenotypes of the AtSAP5 transgenic *Arabidopsis*. The developmental phenotypes of 25-day–old wild-type (WT, Col-0) *Arabidopsis*, AtSAP5 overexpression lines (AtSAP5-oe-4 and AtSAP5-oe-11), and RNAi lines (AtSAP5-RNAi-3 and AtSAP5-RNAi-7). Scale bar, 1 cm.
(TIF)

S9 Fig Expression of immune marker genes in wild-type (WT) and AtSAP5 overexpression and knockdown transgenic *Arabidopsis*. The expression of *NPR1*, *PR1*, *RdR1*, and *GRXC9* in WT, AtSAP5 overexpression (AtSAP5-oe-4, AtSAP5-oe-11), and RNAi lines (AtSAP5-RNAi-3 and AtSAP5-RNAi-7) without treatment were analyzed by qRT-PCR. Data represent mean ± SD; n = 3 biological replicates; significant difference was analyzed by Student’s t-test compared to WT. *Actin* was used as an internal control for normalization.
(TIF)

## References

[1] Pieterse CM, Leon-Reyes A, Van der Ent S, Van Wees SC. Networking by small-molecule hormones in plant immunity. Nat Chem Biol. 2009;5(5):308–16. 10.1038/nchembio.164 . 19377457

[2] White R. Acetylsalicylic acid (aspirin) induces resistance to tobacco mosaic virus in tobacco. Virology. 1979;99(2):410–2. 18631626 10.1016/0042-6822(79)90019-9

[3] Chivasa S, Murphy AM, Naylor M, Carr JP. Salicylic acid interferes with tobacco mosaic virus replication via a novel salicylhydroxamic acid-sensitive mechanism. The Plant Cell. 1997;9(4):547–57. 10.1105/tpc.9.4.547 12237364 PMC156938

[4] Ausubel FM. Are innate immune signaling pathways in plants and animals conserved? Nature Immunology. 2005;6:973–9. 10.1038/ni1253 16177805

[5] Boller T, Felix G. A renaissance of elicitors: Perception of microbe-associated molecular patterns and danger signals by pattern-recognition receptors. Annual Review of Plant Biology. 2009;60:379–406. 10.1146/annurev.arplant.57.032905.105346 19400727

[6] Yang H, Gou X, He K, Xi D, Du J, Lin H, et al BAK1 and BKK1 in Arabidopsis thaliana confer reduced susceptibility to turnip crinkle virus. European journal of plant pathology. 2010;127(1):149–56.

[7] Kørner CJ, Klauser D, Niehl A, Domínguez-Ferreras A, Chinchilla D, Boller T, et al The immunity regulator BAK1 contributes to resistance against diverse RNA viruses. Molecular plant-microbe interactions. 2013;26(11):1271–80. 10.1094/MPMI-06-13-0179-R 23902263

[8] Niehl A, Wyrsch I, Boller T, Heinlein M. Double‐stranded RNAs induce a pattern‐triggered immune signaling pathway in plants. New Phytologist. 2016;211(3):1008–19. 10.1111/nph.13944 27030513

[9] Jones JD, Dangl JL. The plant immune system. Nature. 2006;444(7117):323–9. 10.1038/nature05286 17108957

[10] Fu ZQ, Dong X. Systemic acquired resistance: turning local infection into global defense. Annual review of plant biology. 2013;64:839–63. 10.1146/annurev-arplant-042811-105606 23373699

[11] Cao H, Bowling SA, Gordon AS, Dong X. Characterization of an Arabidopsis mutant that is nonresponsive to inducers of systemic acquired resistance. The Plant Cell. 1994;6(11):1583–92. 10.1105/tpc.6.11.1583 12244227 PMC160545

[12] Delaney T, Friedrich L, Ryals J. Arabidopsis signal transduction mutant defective in chemically and biologically induced disease resistance. Proceedings of the National Academy of Sciences. 1995;92(14):6602–6. 10.1073/pnas.92.14.6602 PMC41566 11607555

[13] Shah J, Tsui F, Klessig DF. Characterization of a s alicylic a cid-i nsensitive mutant (sai1) of Arabidopsis thaliana, identified in a selective screen utilizing the SA-inducible expression of the tms2 gene. Molecular Plant-Microbe Interactions. 1997;10(1):69–78. 10.1094/MPMI.1997.10.1.69 9002272

[14] Cao H, Glazebrook J, Clarke JD, Volko S, Dong X. The Arabidopsis NPR1 gene that controls systemic acquired resistance encodes a novel protein containing ankyrin repeats. Cell. 1997;88(1):57–63. 9019406 10.1016/s0092-8674(00)81858-9

[15] Ryals J, Weymann K, Lavvton K, Friedrich L, Ellis D, Steiner H-Y, et al The Arabidopsis NIM1 protein shows homology to the mammalian transcription factor inhibitor IkB. The Plant Cell. 1997;9(3):425–39. 10.1105/tpc.9.3.425 9090885 PMC156928

[16] Withers J, Dong X. Posttranslational modifications of NPR1: a single protein playing multiple roles in plant immunity and physiology. PLoS pathogens. 2016;12(8):e1005707 10.1371/journal.ppat.1005707 27513560 PMC4981451

[17] Wong CE, Carson RA, Carr JP. Chemically induced virus resistance in Arabidopsis thaliana is independent of pathogenesis-related protein expression and the NPR1 gene. Molecular plant-microbe interactions. 2002;15(1):75–81. 10.1094/MPMI.2002.15.1.75 11858174

[18] Kachroo P, Yoshioka K, Shah J, Dooner HK, Klessig DF. Resistance to turnip crinkle virus in Arabidopsis is regulated by two host genes and is salicylic acid dependent but NPR1, ethylene, and jasmonate independent. The Plant Cell. 2000;12(5):677–90. 10810143 10.1105/tpc.12.5.677 PMC139920

[19] Ding S-W, Voinnet O. Antiviral immunity directed by small RNAs. Cell. 2007;130(3):413–26. 10.1016/j.cell.2007.07.039 17693253 PMC2703654

[20] Yu D, Fan B, MacFarlane SA, Chen Z. Analysis of the involvement of an inducible Arabidopsis RNA-dependent RNA polymerase in antiviral defense. Molecular Plant-Microbe Interactions. 2003;16(3):206–16. 10.1094/MPMI.2003.16.3.206 12650452

[21] Yang S-J, Carter SA, Cole AB, Cheng N-H, Nelson RS. A natural variant of a host RNA-dependent RNA polymerase is associated with increased susceptibility to viruses by Nicotiana benthamiana. Proceedings of the National Academy of Sciences of the United States of America. 2004;101(16):6297–302. 10.1073/pnas.0304346101 15079073 PMC395963

[22] Liu Y, Gao Q, Wu B, Ai T, Guo X. NgRDR1, an RNA-dependent RNA polymerase isolated from Nicotiana glutinosa, was involved in biotic and abiotic stresses. Plant Physiology and Biochemistry. 2009;47(5):359–68. 10.1016/j.plaphy.2008.12.017 19231228

[23] Leibman D, Kravchik M, Wolf D, Haviv S, Weissberg M, Ophir R, et al Differential expression of cucumber RNA‐dependent RNA polymerase 1 genes during antiviral defence and resistance. Molecular plant pathology. 2017. 10.1111/mpp.12518 PMC6637986 27879040

[24] Hunter LJ, Westwood JH, Heath G, Macaulay K, Smith AG, MacFarlane SA, et al Regulation of RNA-dependent RNA polymerase 1 and isochorismate synthase gene expression in Arabidopsis. PLoS One. 2013;8(6):e66530 10.1371/journal.pone.0066530 23799112 PMC3684572

[25] Lee W-S, Fu S-F, Li Z, Murphy AM, Dobson EA, Garland L, et al Salicylic acid treatment and expression of an RNA-dependent RNA polymerase 1 transgene inhibit lethal symptoms and meristem invasion during tobacco mosaic virus infection in Nicotiana benthamiana. BMC plant biology. 2016;16(1):15. 26757721 10.1186/s12870-016-0705-8 PMC4710973

[26] Christenhusz MJ, Byng JW. The number of known plants species in the world and its annual increase. Phytotaxa. 2016;261(3):201–17.

[27] Lu H-C, Hsieh M-H, Chen C-E, Chen H-H, Wang H-I, Yeh H-H. A high-throughput virus-induced gene-silencing vector for screening transcription factors in virus-induced plant defense response in orchid. Molecular Plant-Microbe Interactions. 2012;25(6):738–46. 10.1094/MPMI-10-11-0266 22397405

[28] Giri J, Dansana PK, Kothari KS, Sharma G, Vij S, Tyagi AK. SAPs as novel regulators of abiotic stress response in plants. Bioessays. 2013;35(7):639–48. 10.1002/bies.201200181 23640876

[29] Xie Z, Fan B, Chen C, Chen Z. An important role of an inducible RNA-dependent RNA polymerase in plant antiviral defense. Proceedings of the National Academy of Sciences. 2001;98(11):6516–21. 10.1073/pnas.111440998 PMC33500 11353867

[30] Lai Z, Schluttenhofer CM, Bhide K, Shreve J, Thimmapuram J, Lee SY, et al MED18 interaction with distinct transcription factors regulates multiple plant functions. Nature communications. 2014;5:3064 10.1038/ncomms4064 24451981

[31] Blanco F, Salinas P, Cecchini NM, Jordana X, Van Hummelen P, Alvarez ME, et al Early genomic responses to salicylic acid in Arabidopsis. Plant molecular biology. 2009;70(1–2):79–102. 10.1007/s11103-009-9458-1 19199050

[32] Wertz IE, O'rourke KM, Zhou H, Eby M. De-ubiquitination and ubiquitin ligase domains of A20 downregulate NF-kappaB signalling. Nature. 2004;430(7000):694 10.1038/nature02794 15258597

[33] Tyagi H, Jha S, Sharma M, Giri J, Tyagi AK. Rice SAPs are responsive to multiple biotic stresses and overexpression of OsSAP1, an A20/AN1 zinc-finger protein, enhances the basal resistance against pathogen infection in tobacco. Plant Science. 2014;225:68–76. 10.1016/j.plantsci.2014.05.016 25017161

[34] Vij S, Tyagi AK. A20/AN1 zinc-finger domain-containing proteins in plants and animals represent common elements in stress response. Functional & integrative genomics. 2008;8(3):301–7. 18320246 10.1007/s10142-008-0078-7

[35] Ma A, Malynn BA. A20: linking a complex regulator of ubiquitylation to immunity and human disease. Nature reviews Immunology. 2012;12(11):774 10.1038/nri3313 23059429 PMC3582397

[36] Catrysse L, Vereecke L, Beyaert R, van Loo G. A20 in inflammation and autoimmunity. Trends in immunology. 2014;35(1):22–31. 10.1016/j.it.2013.10.005 24246475

[37] Huang J, Teng L, Li L, Liu T, Li L, Chen D, et al ZNF216 is an A20-like and IκB kinase γ-interacting inhibitor of NFκB activation. Journal of Biological Chemistry. 2004;279(16):16847–53. 10.1074/jbc.M309491200 14754897

[38] Chang E-J, Ha J, Kang S-S, Lee ZH, Kim H-H. AWP1 binds to tumor necrosis factor receptor-associated factor 2 (TRAF2) and is involved in TRAF2-mediated nuclear factor-kappaB signaling. The international journal of biochemistry & cell biology. 2011;43(11):1612–20. 21810480 10.1016/j.biocel.2011.07.010

[39] Kang M, Lee S, Abdelmageed H, Reichert A, Lee HK, Fokar M, et al Arabidopsis stress associated protein 9 mediates biotic and abiotic stress responsive ABA signaling via the proteasome pathway. Plant, cell & environment. 2017;40(5):702–16. 10.1111/pce.12892 28039858

[40] Mattera R, Tsai YC, Weissman AM, Bonifacino JS. The Rab5 guanine nucleotide exchange factor Rabex-5 binds ubiquitin (Ub) and functions as a Ub ligase through an atypical Ub interacting motif and a zinc finger domain. The Journal of Biological Chemistry. 2006;281:6874–83. 10.1074/jbc.M509939200 16407276

[41] Hishiya A, Iemura Si, Natsume T, Takayama S, Ikeda K, Watanabe K. A novel ubiquitin‐binding protein ZNF216 functioning in muscle atrophy. The EMBO Journal. 2006;25(3):554–64. 10.1038/sj.emboj.7600945 16424905 PMC1383529

[42] Miyata N, Okumoto K, Mukai S, Noguchi M, Fujiki Y. AWP1/ZFAND6 Functions in Pex5 Export by Interacting with Cys‐Monoubiquitinated Pex5 and Pex6 AAA ATPase. Traffic. 2012;13(1):168–83. 10.1111/j.1600-0854.2011.01298.x 21980954

[43] Choi H, Han S, Shin D, Lee S. Polyubiquitin recognition by AtSAP5, an A20-type zinc finger containing protein from Arabidopsis thaliana. Biochemical and biophysical research communications. 2012;419(2):436–40. 10.1016/j.bbrc.2012.02.044 22366090

[44] Kang M, Fokar M, Abdelmageed H, Allen RD. Arabidopsis SAP5 functions as a positive regulator of stress responses and exhibits E3 ubiquitin ligase activity. Plant molecular biology. 2011;75(4–5):451–66. 10.1007/s11103-011-9748-2 21293909

[45] Zhang N, Yin Y, Liu X, Tong S, Xing J, Zhang Y, et al The E3 ligase TaSAP5 alters drought stress responses by promoting the degradation of DRIP proteins. Plant physiology. 2017:pp. 01319.2017. 10.1104/pp.17.01319 PMC5717742 29089392

[46] Hymowitz SG, Wertz IE. A20: from ubiquitin editing to tumour suppression. Nature Reviews Cancer. 2010;10:333. 10.1038/nrc2775 20383180

[47] Singh N, Swain S, Singh A, Nandi AK. AtOZF1 Positively Regulates Defense Against Bacterial Pathogens and NPR1-Independent Salicylic Acid Signaling. Molecular Plant-Microbe Interactions. 2018:MPMI-08-17-0208-R. 10.1094/MPMI-08-17-0208-R 29327969

[48] Karimi M, Inzé D, Depicker A. GATEWAY vectors for Agrobacterium-mediated plant transformation. Trends in plant science. 2002;7(5):193–5. 11992820 10.1016/s1360-1385(02)02251-3

[49] Hsing H-X, Lin Y-J, Tong C-G, Li M-J, Chen Y-J, Ko S-S. Efficient and heritable transformation of Phalaenopsis orchids. Botanical studies. 2016;57(1):30 10.1186/s40529-016-0146-6 28597440 PMC5430590

[50] Wu H-Y, Liu K-H, Wang Y-C, Wu J-F, Chiu W-L, Chen C-Y, et al AGROBEST: an efficient Agrobacterium-mediated transient expression method for versatile gene function analyses in Arabidopsis seedlings. Plant methods. 2014;10(1):19. 24987449 10.1186/1746-4811-10-19 PMC4076510

[51] Su C-l, Chen W-C, Lee A-Y, Chen C-Y, Chang Y-CA, Chao Y-T, et al A modified ABCDE model of flowering in orchids based on gene expression profiling studies of the moth orchid Phalaenopsis aphrodite. PLoS One. 2013;8(11):e80462 10.1371/journal.pone.0080462 24265826 PMC3827201

[52] Chang C-SJ, Maloof JN, Wu S-H. COP1-mediated degradation of BBX22/LZF1 optimizes seedling development in Arabidopsis. Plant physiology. 2011;156(1):228–39. 10.1104/pp.111.175042 21427283 PMC3091042

