# Correction: Plant A20/AN1 protein serves as the important hub to mediate antiviral immunity

**DOI:** 10.1371/journal.ppat.1009178

**Published:** 2020-12-22

**Authors:** Li Chang, Ho-Hsiung Chang, Jui-Che Chang, Hsiang-Chia Lu, Tan-Tung Wang, Duen-Wei Hsu, Yuh Tzean, An-Po Cheng, Yi-Shu Chiu, Hsin-Hung Yeh

Additional affiliations for the first and tenth authors are missing. The correct affiliations are as follows:

Li Chang^1,2,3^, Ho-Hsiung Chang^1^, Jui-Che Chang^1^, Hsiang-Chia Lu^4^, Tan-Tung Wang^4^, Duen-Wei Hsu^5^, Yuh Tzean^1^, An-Po Cheng^1^, Yi-Shu Chiu^1^ and Hsin-Hung Yeh^1,2,4,6^

**1** Agricultural Biotechnology Research Center, Academia Sinica, Taipei, Taiwan, **2** Molecular and Biological Agricultural Sciences Program, Taiwan International Graduate Program, National Chung Hsing University and Academia Sinica, Taipei, Taiwan, **3** Graduate Institute of Biotechnology, National Chung Hsing University, Taichung, Taiwan, **4** Department of Plant Pathology and Microbiology, National Taiwan University, Taipei, Taiwan, **5** Department of Biotechnology, National Kaohsiung Normal University, Kaohsiung, Taiwan, **6** Biotechnology Center, National Chung Hsing University, Taichung, Taiwan.
