# Supplementary material for: Plant A20/AN1 protein serves as the important hub to mediate antiviral immunity
Source: PLoS Pathog. 2018 Sep 13;14(9):e1007288. doi: 10.1371/journal.ppat.1007288 (PMC6155556; doi:10.1371/journal.ppat.1007288)
Supplement: S1 Table — (PDF) [file ppat.1007288.s001.pdf]

S1 Table. *Pha13*-responsive differentially expressed genes (DEGs) compared to vector control are classified into NPR1-dependent and -independent groups following previous studies.

ID[a]	Alias	Description	*Arabidopsis* Homolog[b]	P value[c]	Fold changes[c]	References
NPR1-dependent genes						
PATC145800	WRKY70	WRKY Transcription Factor	At3g56400	0.000010	14.11	[53]
PATC152659	WRKY53	WRKY Transcription Factor	At4g23810	0.000282	5.19	[53]
PATC139697	Protein kinase	Protein kinase superfamily protein	At3g09010	0.013400	1.63	[31]
PATC148973	APK2b	Protein kinase 2B	At2g02800	0.014900	2.05	[31]
PATC141782	YSL3	YELLOW STRIPE like 3	At5g53550	0.000426	3.11	[31]
PATC135791	NPR1	Key regulator of the salicylic acid (SA)-mediated systemic acquired resistance (SAR) pathway.	At1g64280	0.014900	1.61	
PATC154483		LisH and RanBPM domain-containing protein	AT1g61150	0.006653064	1.44	[53]
PATC133682	LURP1	A member of the LURP cluster which exhibits pronounced upregulation after recognition of the pathogenic oomycte *H. parasitica.*	AT2g14560	0.000362737	2.38	[53]
PATC142626	CPFTSY	Chloroplast SRP receptor homolog. Required for LHCP integration into isolated thylakoids.	AT2g45770	0.04253212	1.38	[53]

S1 Table. Cont.

PATC154050		Encodes an aspartic protease, has an important regulatory function in chloroplasts.	AT3g12700	0.000322238	3.64	[53]
PATC143146	RdR1	RNA-dependent RNA polymerase	At1g14790	0.031000	1.38	
NPR1-independent genes						
PATC152473	SSI2	A stearoyl-ACP desaturase, involved in fatty acid desaturation	At2g43710	0.019400	1.34	[54]
PATC125646	WRKY3	WRKY DNA-binding protein 3	At2g03340	0.001050	2.03	[55]
PATC129486	WRKY21	WRKY DNA-binding protein 21	At2g30590	0.003180	1.57	[55]
PATC154824	IEGT	UDP-glucosyl transferase 73B1	AT4g34138	0.000117	2.32	[56]
PATC068819	GRXC9	Encodes GRX480, a member of the glutaredoxin family that regulates protein redox state	At1g28480	0.000002	15.04	[57]
PATC132182	GRXS13	Glutaredoxin ATGRXS13, required to facilitate *Botrytis cinerea* infection of *Arabidopsis thaliana* plants.	At1g03850	0.006730	1.9	[57]
PATC148347	ADH1	Catalyzes the reduction of acetaldehyde using NADH as reductant	At1g77120	0.002740	1.67	[31]
PATC225126	DMR6	DOWNY MILDEW RESISTANT 6. Encodes a putative 2OG-Fe(II) oxygenase that is defense-associated but required for susceptibility to downy mildew	At5g24530	0.015500	2.06	[31]

S1 Table. Cont.

PATC143725	ASPGB1	Encodes an asparaginase that catalyzes the degradation of L-asparagine to L-aspartic acid and ammonia.	At3g16150	0.001380	3.23	[53]
PATC139952	ATAPR3	A member of a multigene family within the thioredoxin (TRX) superfamily.	At4g21990	0.000346	3.47	[31]
PATC156665	ANAC002	A member of the NAC family of transcription factors	At1g01720	0.000008	6.48	[31]
PATC127565	AOX	The alternative oxidase of plant mitochondria transfers electrons from the ubiquinone pool to oxygen without energy conservations	At3g22370	0.039800	1.25	[53]

[a]Accession number of Orchidstra database (http://orchidstra.abrc.sinica.edu.tw)

[b]Accession number of TAIR (http://www.Arabidopsis.org)

[c]All DEGs are selected based on significance (unpaired t test $P < 0.05$)
