# Supplementary material for: Plant A20/AN1 protein serves as the important hub to mediate antiviral immunity
Source: PLoS Pathog. 2018 Sep 13;14(9):e1007288. doi: 10.1371/journal.ppat.1007288 (PMC6155556; doi:10.1371/journal.ppat.1007288)
Supplement: S2 Table — (PDF) [file ppat.1007288.s002.pdf]

S2 Table. Pha13 interacting proteins identified by yeast-two-hybrid screening

ID[a]	Alias	Description	*Arabidopsis* Homolog[b]	Number of clones
PATC155725	UBQ10	One of five polyubiquitin genes in *A. thaliana*	AT4G05320	20
PATC156673	UBQ4	Encodes a ubiquitin polyprotein.	AT5G20620	4
PATC233706	UBQ13	Polyubiquitin gene	AT1G65350	1
PATC125694	DNAJ homolog	DNAJ heat shock family protein involved in protein folding	AT2G20560	2
PATC143280	Cytosolic thioredoxin	Encodes a cytosolic thioredoxin that reduces disulfide bridges of target proteins by the reversible formation of a disulfide bridge between two neighboring Cys residues present in the active site.	AT3G51030	2
PATC156413	Cytosolic thioredoxin	Encodes a cytosolic thioredoxin that reduces disulfide bridges of target proteins by the reversible formation of a disulfide bridge between two neighboring Cys residues present in the active site.	AT3G51030	2
PATC154796	A20/AN1 ZF	A20/AN1-like zinc finger family protein; Function in DNA binding, zinc ion binding	AT2G36320	1

S2 Table. Cont.

PATC146799	FD3	Ferredoxin 3 (FD3) involved in electron transport chain	AT2G27510	1
PATC150760	WD40 repeat-like protein	Transducin/WD40 repeat-like superfamily protein	AT4G18905	1
PATC126521	Dihydrolipoamide succinyltransferase	Dihydrolipoamide succinyltransferase; Function in zinc ion binding, acyltransferase activity.	AT4G26910	1
PATC130336	SLOMO F-Box	Encodes SLOMO (SLOW MOTION), an F-box protein required for auxin homeostasis and normal timing of lateral organ initiation at the shoot meristem.	AT4G33210	1
PATC146324	TRAP	Translocon-associated protein (TRAP), alpha subunit	AT2G21160	1
PATC147016	DNAJ homolog	Homologous to the co-chaperon DNAJ protein from *E. coli*	AT3G44110	1
PATC142935	LOS1	LOW EXPRESSION OF OSMOTICALLY RESPONSIVE GENES 1 encodes a translation elongation factor 2-like protein that is involved in cold-induced translation.	AT1G56070	1

S2 Table. Cont.

PATC129205	SWAP70	Pleckstrin homology (PH) domain-containing protein involved in signal transduction	AT2G30880	1
PATC130549	APT3	Adenine phosphoribosyl transferase 3	AT4G22570	1
PATC157121	Alpha/beta hydrolase	Involved in the lipid metabolic process	AT3G14360	1
PATC140414	OB-fold like protein	Nucleic acid-binding, OB-fold-like protein; Function in RNA binding, translation initiation factor activity	AT2G04520	1
PATC137737	Copia-like retrotransposon family	Copia-like retrotransposon family	AT1G31210	1
PATC157709	ELIP	Encodes an early light-inducible protein	AT3G22840	1
PATC155095	SABATH methyltransferase	A member of the *Arabidopsis* SABATH methyltransferase gene family. Encodes NAMT1, a methyltransferase that methylates nicotinic acid.	AT5G04370	1

S2 Table. Cont.

PATC155413	EF1a, Tu family	GTP binding elongation factor Tu family protein; Function in calmodulin binding, translation elongation factor activity; Involved in translational elongation	AT5G60390	1
PATC131101	HAD superfamily protein	Haloacid dehalogenase-like hydrolase (HAD) superfamily protein; Function in phosphatase activity	AT3G55960	1
PATC140021	Aldolase-type TIM barrel family protein	Aldolase-type TIM barrel family protein	AT4G02610	1
PATC157778	RBP L18	Encodes cytoplasmic ribosomal protein L18	AT3G05590	1
PATC228024	Peroxisomal catalase	Loss of function mutations have increased H2O2 levels and increased H2O2 sensitivity.	AT4G35090	1
PATC126060	Chloroplast-localized pentatricopeptide-repeat protein	Encodes a chloroplast-localized pentatricopeptide-repeat protein involved in regulation of nuclear gene expression.	AT2G31400	1

S2 Table. Cont.

PATC125551	Mitochondrial substrate carrier family protein	Mitochondrial substrate carrier family protein; Functions in binding, transporter activity; Involved in transport, mitochondrial transport, transmembrane transport	AT3G55640	1
PATC129335	Serinc-domain containing serine and sphingolipid biosynthesis protein		AT1G16180	1

[a]Accession number of Orchidstra database (http://orchidstra.abrc.sinica.edu.tw)

[b]Accession number of TAIR (http://www.Arabidopsis.org)
