# Supplementary material for: Plant A20/AN1 protein serves as the important hub to mediate antiviral immunity
Source: PLoS Pathog. 2018 Sep 13;14(9):e1007288. doi: 10.1371/journal.ppat.1007288 (PMC6155556; doi:10.1371/journal.ppat.1007288)
Supplement: S3 Table — (PDF) [file ppat.1007288.s003.pdf]

S3 Table. Primers and probes used in this study

Name of primers and probes	Nucleotide sequence	Description
**Semi-quantitative RT-PCR**		
CymMV-F	5'-GAAATAATCATGGGAGAGCC-3'	Detection of *Cymbidium*
CymMV-R	5'-AGTTTGGCGTTATTCAGTAGG-3'	*mosaic virus* (CymMV)
ORSV-F	5'-ACGCACAATCTGATCCGTA-3'	Detection of *Odontoglossum*
ORSV-R	5'-ATCCGCAGTGAAAACCC-3'	*ringspot virus* (ORSV)
Pha13-F	5'-TCATCTTAAAACAGAGCAGGATCTGA-3'	Detection of *Pha13*
Pha13-R	5'-ATTGGCCTGGACCACCGGGTT-3'	(PATC148746[a])
PhaRdR1-F	5'-GCACGGGAGCTACGAAAGTCAA AT-3'	Detection of *PhaRNA-dependent RNA*
PhaRdR1-R	5'-TTAATGGGCAGCTGCAAACAAATC-3'	*polymerase 1* (*PhaRdR1*; PATC143146[a])
PhaGRX-F	5'-TCATTATCTTCTTCCTTCGGCGG-3'	Detection of *PhaGlutaredoxin*
PhaGRX-R	5'-CTACAACCACAGAGCTCCAGCCTC-3'	*C9* (*PhaGRX*; PATC068819[a])
PhaUBQ 10-F	5'-TACCGGTAAGACTTTCAACCTCT-3'	Detection of *PhaUbiquitin 10*
PhaUBQ 10-R	5'-AAGGCCAAAATTCAGGATAAAGAG-3'	(*PhaUBQ10*; PATC230548[a])
PhaPR1-F	5'-TGCGAGGCTGAATTAACAACGAA-3'	Detection of *PhaPathogenesis-*
PhaPR1-R	5'-GCCAGGAACATTACCAGGAGGATA-3'	*related protein 1* (*PhaPR1*; PATC126136[a])
**Real-time RT-PCR**		
Pha13-qF	5'-TCATCTTAAAACAGAGCAGGATCTGA-3'	Detection of *Pha13*
Pha13-qR	5'-CGGCGTCTAAAGAAGAGGAAGAG-3'	
PhaNPR1-qF	5'-AGTTGATGGCACCTTGGAGTTT-3'	Detection of *PhaNonexpressor*
PhaNPR1-qR	5'-AAGAAGCGTTTCCCGAGTTCA-3'	*of pathogenesis-related protein 1* (*PhaNPR1*; PATC135791[a])
PhaPR1-qF	5'-GGATCATCGTCTTGCGATTT-3'	Detection of *PhaPR1*
PhaPR1-qR	5'-CCGCACAACTGTTACATGCAT-3'	
CymMV-qF	5'-TGATGCTGGCCACTAACGATC-3'	Detection of CymMV
CymMV-qR	5'-GGAATCAACGGCATCGAAGA-3'	
PhaRdR1-qF	5'-TGACAAGTACAAGGATGGCG-3'	Detection of *PhaRdR1*
PhaRdR1-qR	5'-ACTCTTCACCCCATCAACATC-3'	
PhaGRX-qF	5'-ACGTCGTAAAGCATCTCCTT-3'	Detection of *PhaGRX*
PhaGRX-qR	5'-CGGTAAGGTCACCGTAAAAC-3'	

S3 Table. Cont.

PhaUBQ 10-qF<br>PhaUBQ 10-qR	5'-CCGGATCAGCAAAGGTTGA-3'<br>5'-AAGATTTGCATCCCTCCCC-3'	Detection of *PhaUBQ10*
PhaJAZ1-qF<br>PhaJAZ1-qR	5'-CTCGGAGTGGTTGGCTTTATTAG-3'<br>5'-GTGATGCCTTCCTTGCGATT-3'	Detection of *PhaJasmonate ZIM-domain protein 1* (*PhaJAZ1*; PATC141437[a])
PhaACO2-qF<br>PhaACO2-qR	5'-CGTCCAAAACGCTTGATTCC-3'<br>5'-GCGCAAATTCCTTCATGGTT-3'	Detection of *PhaAmino-cyclopropane-carboxylate oxidase 2* (*PhaACO2*; PATC139319[a])
AtACT-qF<br>AtACT-qR	5'-GGCAAGTCATCACGATTGG -3'<br>5'-CAGCTTCCATTCCCACAAAC -3'	Detection of *AtActin* (At3G18780)
TRV-qF<br>TRV-qR	5'-CATGCTAACAAATTGCGAAAGC-3'<br>5'-CACAGACAAACCATCCACAATTATTTT-3'	Detection of TRV
CMV-qF<br>CMV-qR	5'-TTCCTGCCTCCTCGGACTTA-3'<br>5'-GCTCCGTCCGCGAACATA-3'	Detection of CMV
AtSAP5-qF<br>AtSAP5-qR	5’-ACCAGCTAAAGTCGTGATTCG-3’<br>5’-AGCGGTTTTGTAGTCGTAGC-3’	Detection of *AtSAP5*
AtNPR1-qF<br>AtNPR1-qR	5’-AGGAGATTGCCAAGGATTACG-3’<br>5’-ACGTCCAATAAGTGCCTCTG-3’	Detection of *AtNPR1*
AtPR1-qF<br>AtPR1-qR	5’-ATCGTCTTTGTAGCTCTTGTAGG-3’<br>5’-AGGCTAAGTTTTCCCCGTAAG-3’	Detection of *AtPR1*
AtRdR1-qF<br>AtRdR1-qR	5’-AAGAGCGGTTCGGGCGTTGA-3’<br>5’-AGCCGAAGCCTTTGCTGACTCA-3’	Detection of *AtRdR1*
AtGRXC9-qF<br>AtGRXC9-qR	5’-AGAGAGTTCGGATGGTGGTG-3’<br>5’-CACTCCAAGTCCAAGAAGCAG-3’	Detection of *AtGRXC9*
**Construction**		
attB1-Pha13-F[b]<br>attB2-Pha13-R[b]	5'-GGGGACAAGTTTGTACAAAAAAGCAGGCTCGTGGAGAAATCCCTCATGGCT-3'<br>5'-GGGGACCACTTTGTACAAGAAAGCTGGGTTCAAATCTTCCGCAGCTTATCG-3'	Construction of pCymMV-Pha13-virus induced gene silencing vector
Pha13-hpRNA-F<br>Pha13-hpRNA-R	5'-CACCCCGGTCCAACCCGGTGGTCCA-3'<br>5'-TGGACCACCGGGTTGGACCGG-3'	Construction of phpPha13
PhaNPR1-hpRNA-F<br>PhaNPR1-hpRNA-R	5' -CACCCGAGATCATTGATTCCTTCCC-3'<br>5' -GGGAAGGAATCAATGATCTCG-3'	Construction of phpPhaNPR1

S3 Table. Cont.

PhaRdR1-hpRNA-F	5' -CACCGCCTATCACTGCGTCACATGT-3'	Construction of phpPhaRdR1
PhaRdR1-hpRNA-R	5' -ACATGTGACGCAGTGATAGGC-3'	
PhaGRX-hpRNA-F	5' -CACCGCTAATGGCGGCGCATATTAC-3'	Construction of phpPhaGRX
PhaGRX-hpRNA-R	5' -GTAATATGCGCCGCCATTAGC-3'	
Pha13-ORF-F	5'-CACCATGGCGGAAGAACGGAGATG-3'	Construction of pG-Pha13 or pPha13-G
Pha13-ORF-NONSTOP-R	5'-AATCTTCCGCAGCTTATCGGCC-3'	
FLAG-Pha13ORF-F	5'-CACCATGGATTACAAGGATGACGACGATAAGATGGCGGAAGAACG-3'	Construction of pPha13-oe and pHPha13
Pha13ORF-R	5'-TCAAATCTTCCGCAGCTTATCGG-3'	
AtSAP5-RNAi-F	5'- CACCCGCAGGTGTTGTTGAATCAGG-3'	Construction of phpAtSAP5-RNAi
AtSAP5-RNAi-R	5'- GATCTGTTGATCTCTCTTCTT-3'	
AtSAP5ORF-F	5'- CACCATGGCTCAGAGAACGGAGAAG-3'	Construction of pAtSAP5-oe
AtSAP5ORF-HA-R	5'-TCAAGCGTAATCTGGAACATCGTATGGGTAAACTTTGACCATTTTCGCAGC-3'	
Pha13A20m-F	5'-GCAACCCTAAACCTCGGCTCGAGCGGCTACCGCGATCATC -3'	Construction of pPha13A20m
Pha13A20m-R	5'-GATGATCGCGGTAGCCGCTCGAGCCGAGGTTTAGGGTTGC-3'	
Pha13AN1m-F	5'-CTGACCGGGTTCAAGGGCCGTTGCGGATCGACGTACTGTGGGGCCGGCCGGTACCCGGAGCA-3'	Construction of pPha13AN1m
Pha13AN1m-R	5'-TGCTCCGGGTACCGGCCGGCCCCACAGTACGTCGATCCGCAACGGCCCTTGAACCCGGTCAG-3'	
NdeI-Pha13-F[c]	5'-TTCTCATATGGCGGAAGAACGGAGATGCCAA-3'	Construction of pETPha13, pETPha13A20m, pETPha13AN1m and pETPha13A20mAN1m
NotI-Pha13-R[c]	5'-ATATGCGGCCGCAATCTTCCGCAG-3'	
NdeI-Pha13-F[c]	5'-AGAGCATATGGCTGCCACCTTCTCCTCCTTTT-3'	Construction of pGBKPha13
EcoR1-Pha13-R[c]	5'-AGAGGAATTCAAATCTTCCGCAGCTTA-3'	

S3 Table. Cont.

Nd13Fs412F[c]	5’-CTAACATATGCGGGATTCGATTGCC-3’	
Nd13Ap361F[c]	5’-CTAACATATGCACCGGTACCCGGAG-3’	For construction of Pha13 with N-terminus deletions into pGBK
Nd13Sg247F[c]	5’CTAACATATGCCGGCGGTCGCCGAT-3’	
Nd13Bt136F[c]	5’CTAACATATGGTGGAGAAATCCCTC-3’	
Nd13Ps88F[c]	5’CTAACATATGTCGAGCTGCTACCGC-3’	
EcoR1-Pha13-R[c]	5'-AGAGGAATTCAAATCTTCCGCAGCTTA-3'	
Nd13A20F[c]	5’-CTAACATATGCAAGAAGGTATGCGA-3’	For construction of A20 domain into pGBK
BH13A20R[c]	5’-CTAAGGATCCAAGATGATCGCGGTA-3’	
Nd13AN1F[c]	5’-CTAACATATGGAGAGGCCGAGAAGG-3’	For construction of AN1 domain into pGBK
BH13AN1R[c]	5’-CTAAGGATCCCTTATAGTCGAAATC-3’	
NdeI-PhaUBQ-F[c]	5´-CTCTCATATGAGGGGAGGTATGCAGATTTTCG-3'	Construction of polyubiquitin (PATC203893)
BamHI-PhaUBQ-R[c]	5'-CTATGGATCCACCACGAAGACGGAGCACAA-3'	
NdeI-AtSAP5-F[c]	5’-TTCTCATATGATGGCTCAGAGAACGGAGAAG-3’	Construction of pETAtSAP5
XhoI-AtSAP5-R[c]	5’- AGAGCTCGAGAACTTTGACCATTTTCGCAGC-3’	

[a]Accession number of Orchidstra database (http://orchidstra.abrc.sinica.edu.tw)

[b]Underlined sequences indicate the attB sequences. The 4 G at the 5' end were added as recommended by the manufacturer.

[c]Underlined sequences indicate the sequence recognized by restriction enzyme. The random 4 nucleotides at the 5' end were added as recommended by the manufacture
