# Supplementary material for: Plant A20/AN1 protein serves as the important hub to mediate antiviral immunity
Source: PLoS Pathog. 2018 Sep 13;14(9):e1007288. doi: 10.1371/journal.ppat.1007288 (PMC6155556; doi:10.1371/journal.ppat.1007288)
Supplement: S1 File — (PDF) [file ppat.1007288.s004.pdf]

The sequence of Pha13 coding region

Pha13 (PATC148746)

ATGGCGGAAGAACGGAGATGCCAAGAAGGTATGCGATGCGCGAACAACT
GCGGTTTCTTCGGCAGCCCTGCAACCCTAAACCTCTGCTCGAGCTGCTACC
GCGATCATCTTAAAACAGAGCAGGATCTGATCGCCGTGGAGAAATCCCTC
ATGGCTGCCACCTTCTCCTCCTCCTCCTCCTCTTCCTCTTCTTTAGACG
CCGTGCTTTCCTCCTCTCCTCCCTTCTCCGCCGCCGCGTGTGCACCGGCGG
TCGCCGATGAGCCGGCGGAGGAGCGGCCGAGAAGGTGCATGGCTTGCCG
GAAAAAGGTGGGGCTGACAGGGTTCAAGTGCCGTTGCGGATCGACGTTCT
GTGGGGCCCACCGGTACCCGGAGCATCACGCCTGCGATTTCGACTATAAG
TCGGCCGGCCGGGATTCGATTGCCCGGTCCAACCCGGTGGTCCAGGCCGA
TAAGCTGCGGAAGATTTGA
