# Supplementary figures and images for: Plant A20/AN1 protein serves as the important hub to mediate antiviral immunity
Source: PLoS Pathog. 2018 Sep 13;14(9):e1007288. doi: 10.1371/journal.ppat.1007288 (PMC6155556; doi:10.1371/journal.ppat.1007288)

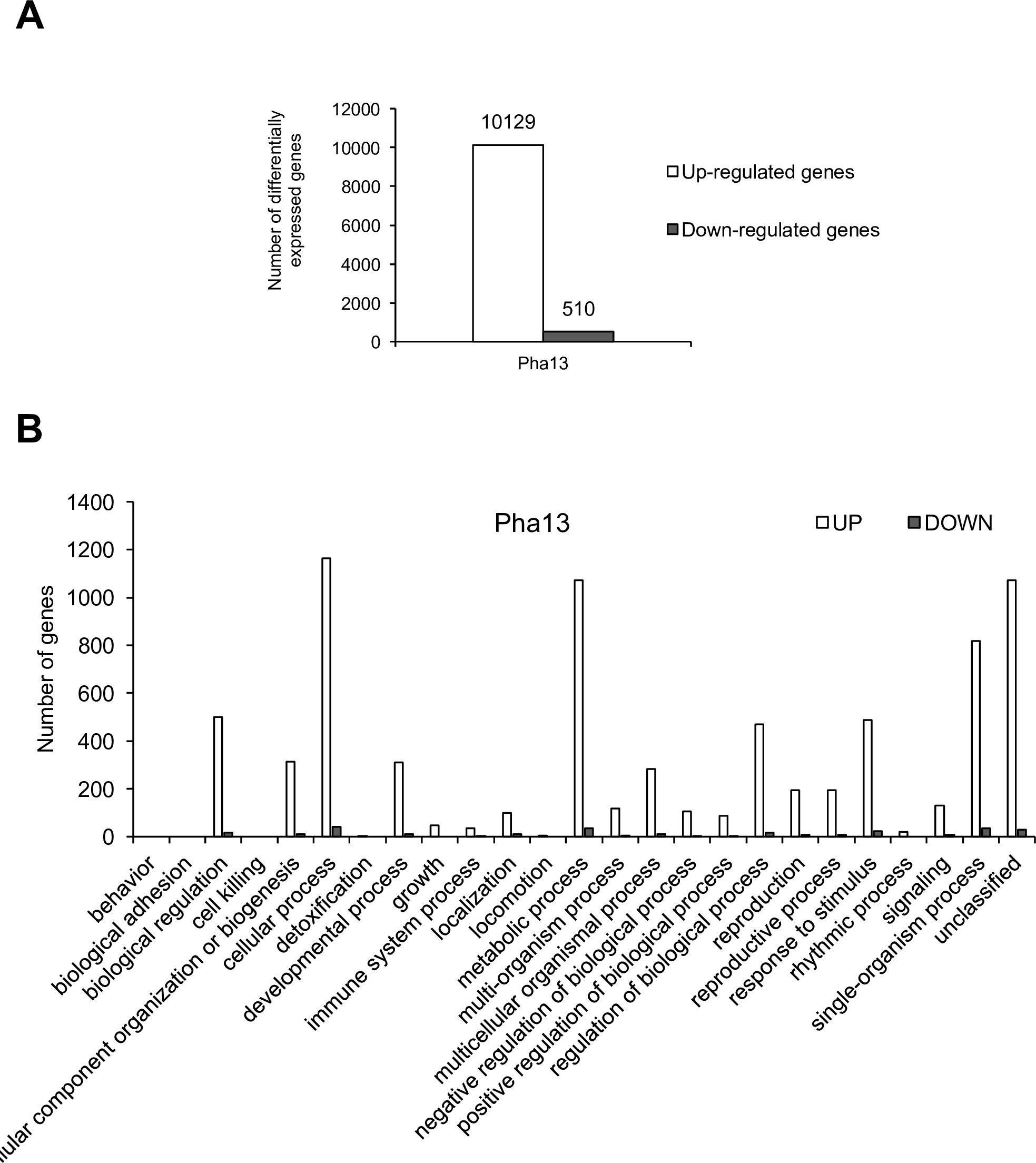

Supplement: S1 Fig — (A) The number of up- or down-regulated differentially expressed genes (DEGs) in Pha13 transiently overexpressed leaves of P. aphrodite. (B) Gene Ontology (GO) analysis of up- and down-regulated DEGs in Pha13 transiently overexpressed leaves of P. aphrodite. The DEGs are classified into subcategories of biological process GO terms (X-axis). The Y-axis represents the number of genes. (TIF) [file ppat.1007288.s005.tif]

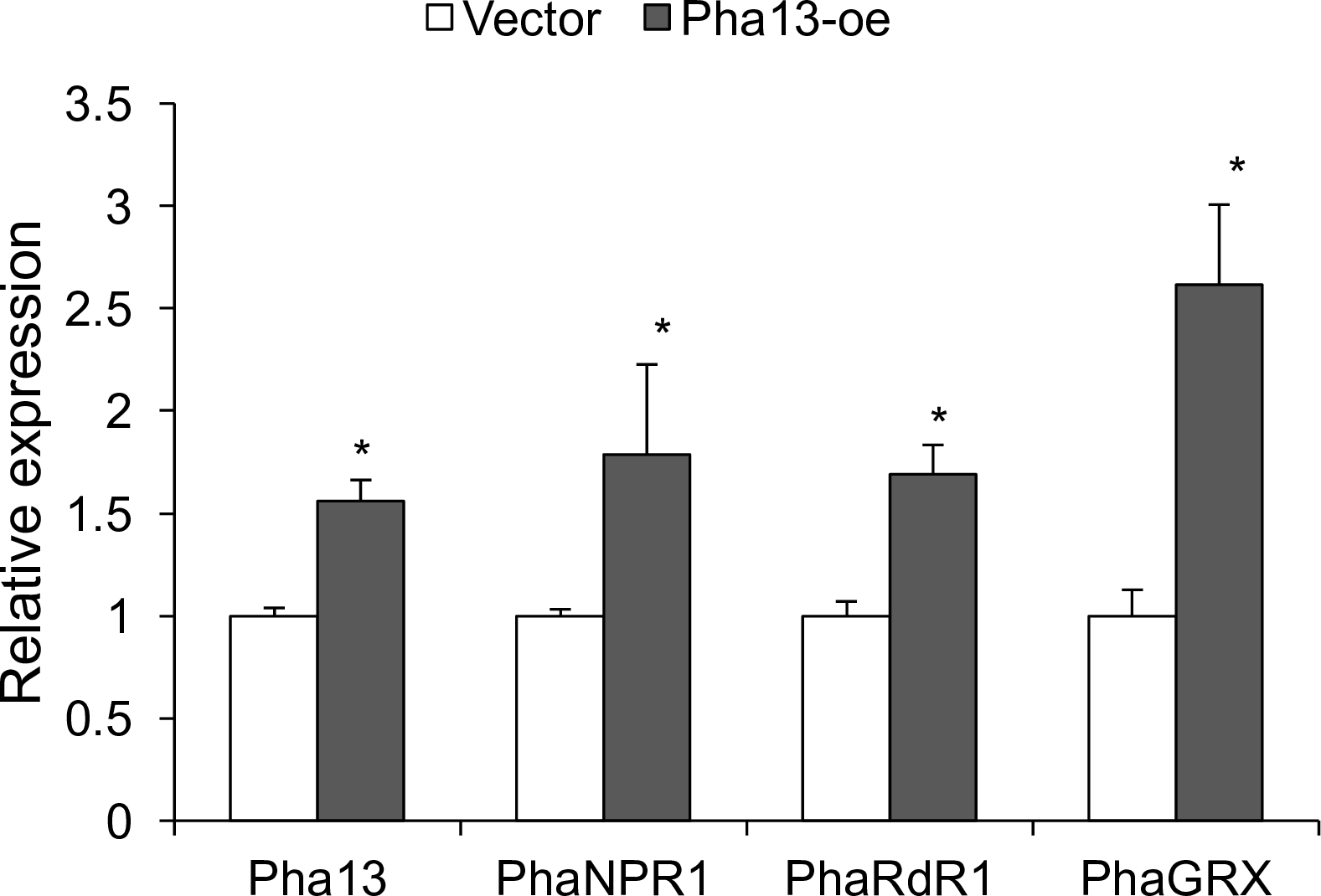

Supplement: S2 Fig — Expression level of Pha13, PhaNPR1 PhaRdR1, and PhaGRX were analyzed by qRT-PCR from leaves of P. aphrodite infiltrated with agrobacterium carrying vector (Vector), or plasmid to overexpress Pha13 (Pha13-oe). The RNA level of vector was set to 1. Data represent mean ± SD; n = 3 biological replicates; *, P < 0.05, Student’s t-test compared to vector. PhaUbiquitin 10 was used as an internal control for normalization. (TIF) [file ppat.1007288.s006.tif]

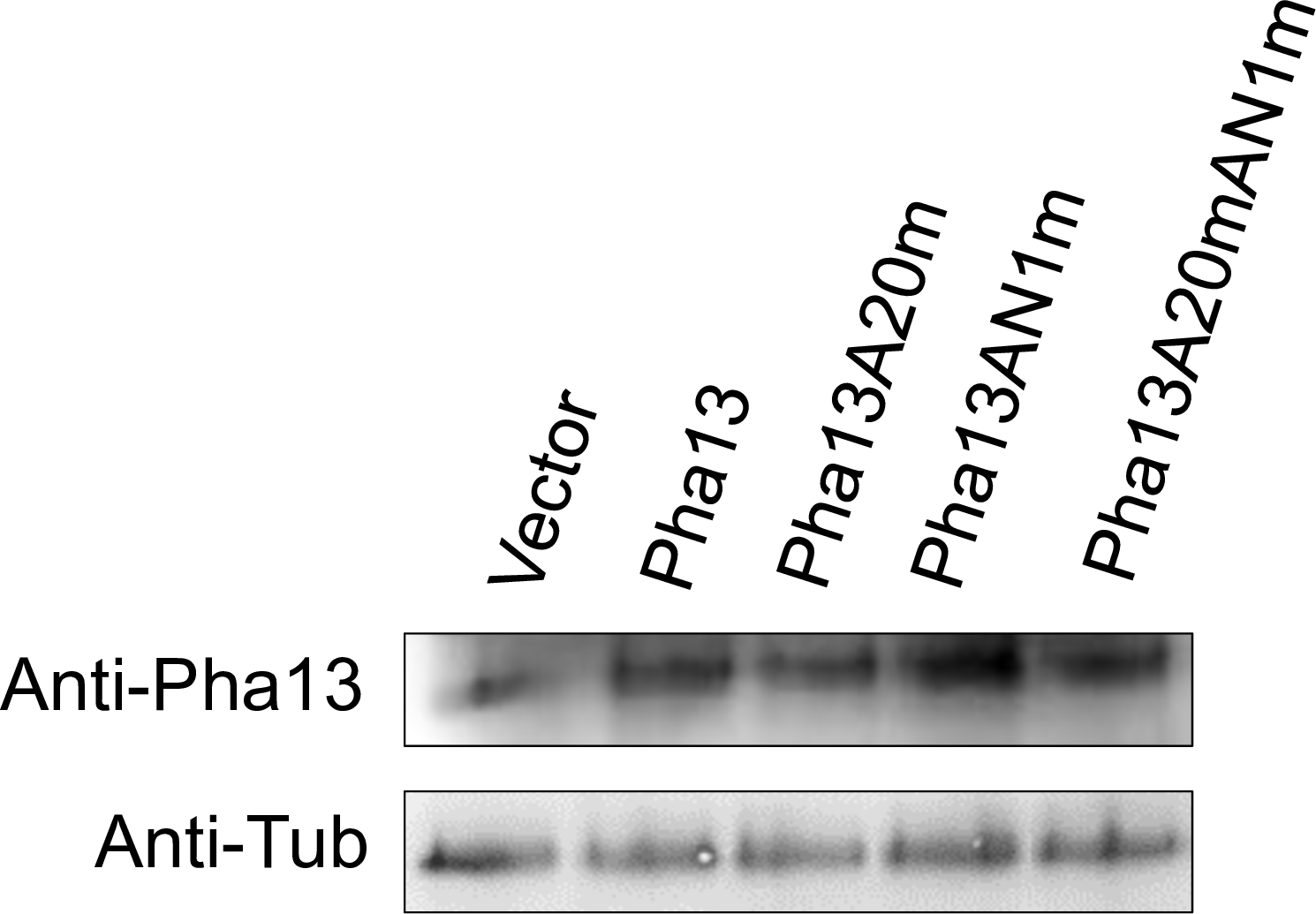

Supplement: S3 Fig — Leaves of P. aphrodite was infiltrated with agrobacterium carrying vector (pK2GW7), overexpression clones of wild-type Pha13 (pPha13-oe), or the respective A20 and/or AN1 mutant clone (pPha13A20m, pPha13AN1m or pPha13A20mAN1m). Total proteins extracted from the infiltrated leaves were used for immunoblotting analysis with the use of anti-Pha13 antibody (Anti-Pha13). The anti-tubulin antibody (Anti-Tub) was used as a loading control. (TIF) [file ppat.1007288.s007.tif]

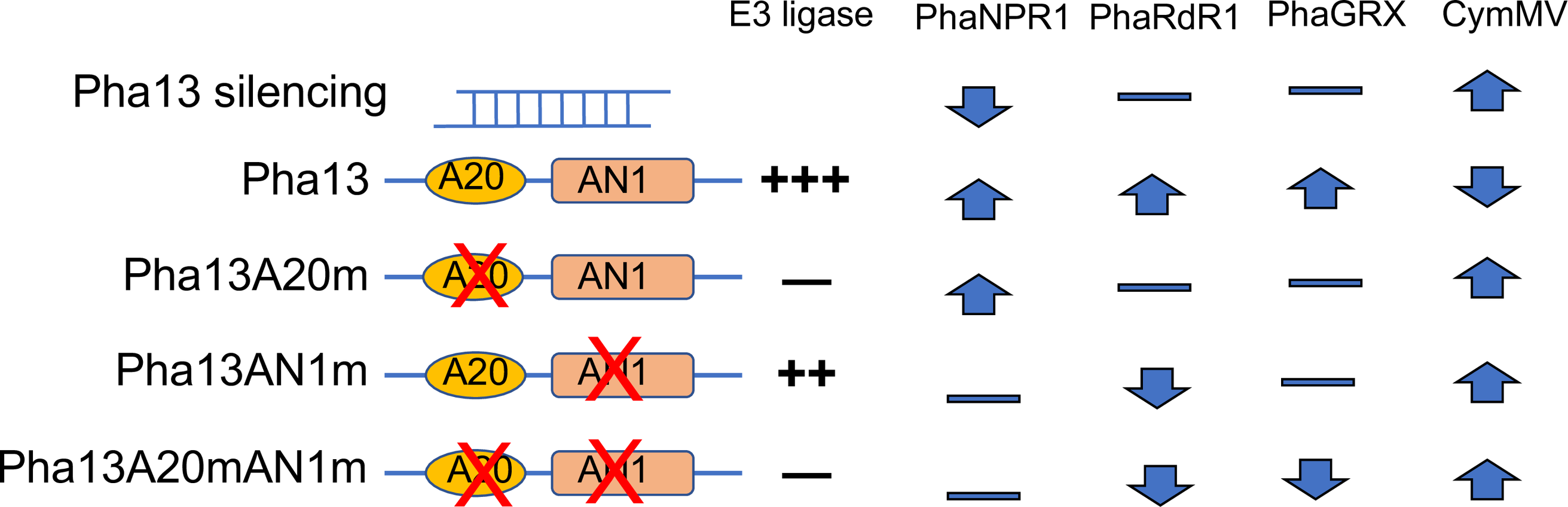

Supplement: S4 Fig — Pha13 E3 ligase activity and effects on expression of PhaNPR1, PhaRdR1, PhaGRX, and accumulation level of CymMV. Silencing and overexpression of wild-type Pha13 or derived mutant clones (Pha13A20m, Pha13AN1m, and Pha13A20mAN1m) in P. aphrodite are depicted. The oval circle and rectangle indicate the A20 and AN1 domain, respectively. The “X” symbol indicates the mutated A20 and/or AN1 domain(s). The strength of E3 ligase activity is indicated with “+” or “-”. Gene(s) -up and -down regulation is indicated with blue arrows pointing up and down, respectively. Unaffected gene expression is indicated with a horizontal blue line. (TIF) [file ppat.1007288.s008.tif]

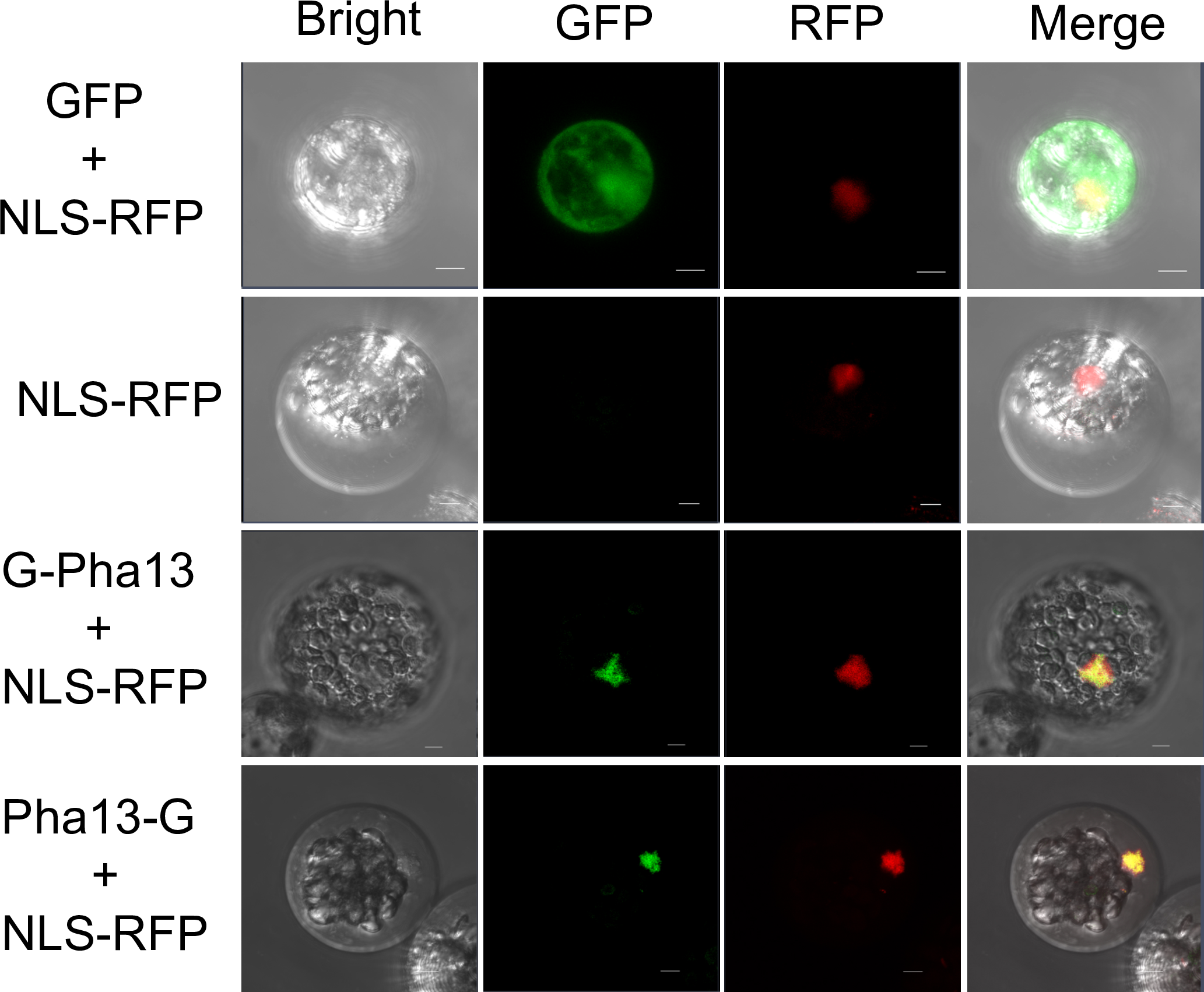

Supplement: S5 Fig — Green fluorescent protein (GFP), N- and C- terminal GFP-fused Pha13 (G-Pha13 and Pha13-G) were transfected either alone or co-transfected with nucleus localization signal fused red fluorescence protein (NLS-RFP) in protoplasts of P. aphrodite. Fluorescence was detected by confocal microscopy after transfection. Scale bars represent 10 μm. (TIF) [file ppat.1007288.s009.tif]

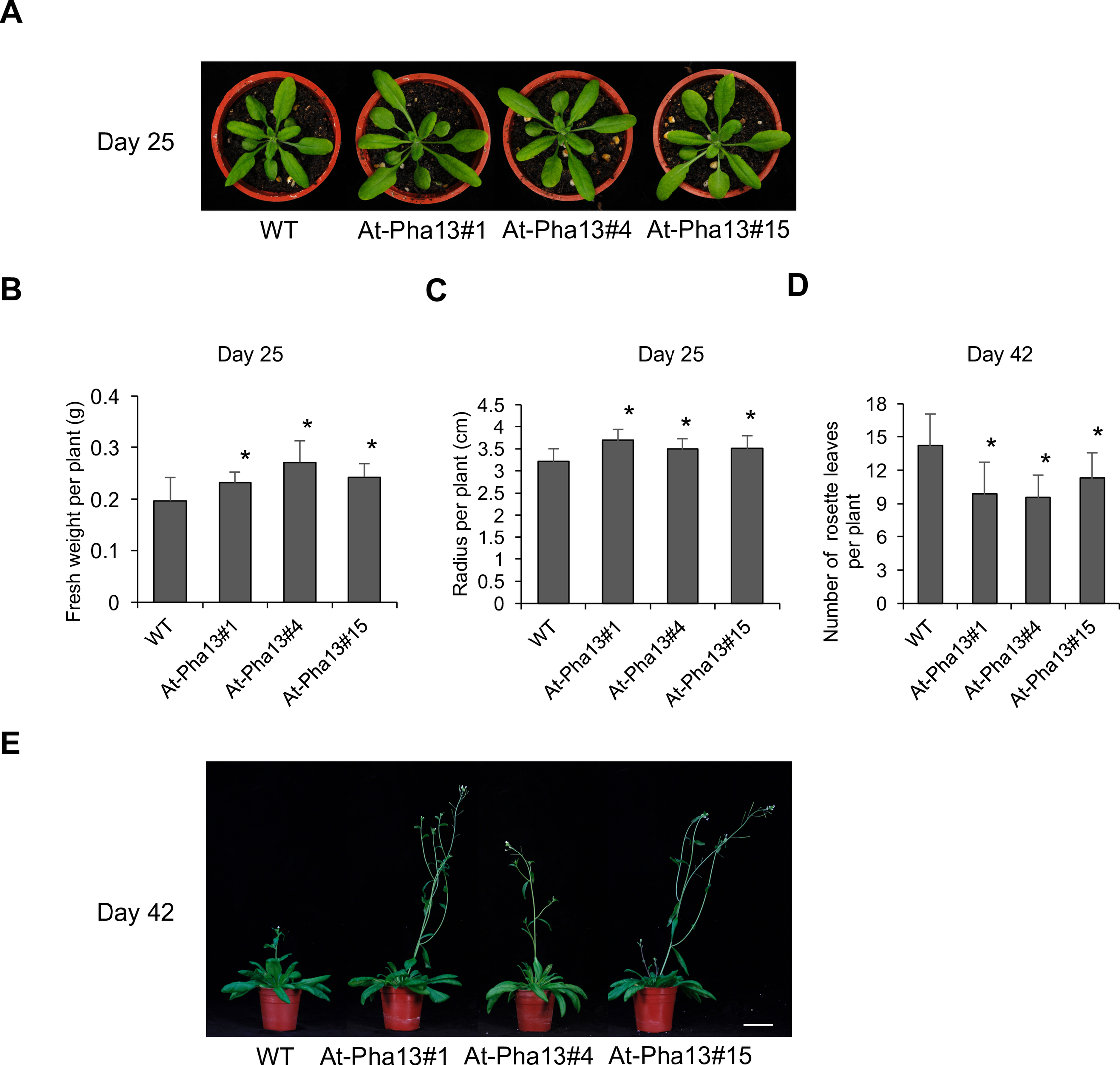

Supplement: S6 Fig — (A to C) The developmental phenotypes of 25-day–old T3 progenies (35S∷FLAG-Pha13) derived from three T1 transgenic lines, At-Pha13#1, At-Pha13#4, and At-Pha13#15 were presented on A. Shoot fresh weight (B) and radius (C) of the plants were analyzed. (D and E) The total number of rosette leaves of 42-day-old T3 progenies (35S∷FLAG-Pha13) were measured (D) and photos of representative plants are shown on (E). A-E. On A, scale bar, 1 cm. For B and C, data represent mean ± SD; n = 7 biological replicates; *, P < 0.05, Student’s t-test compared to WT. For D, data represent mean ± SD; n = 10 biological replicates; *, P < 0.05, Student’s t-test compared to WT. On E, scale bar, 5 cm. (TIF) [file ppat.1007288.s010.tif]

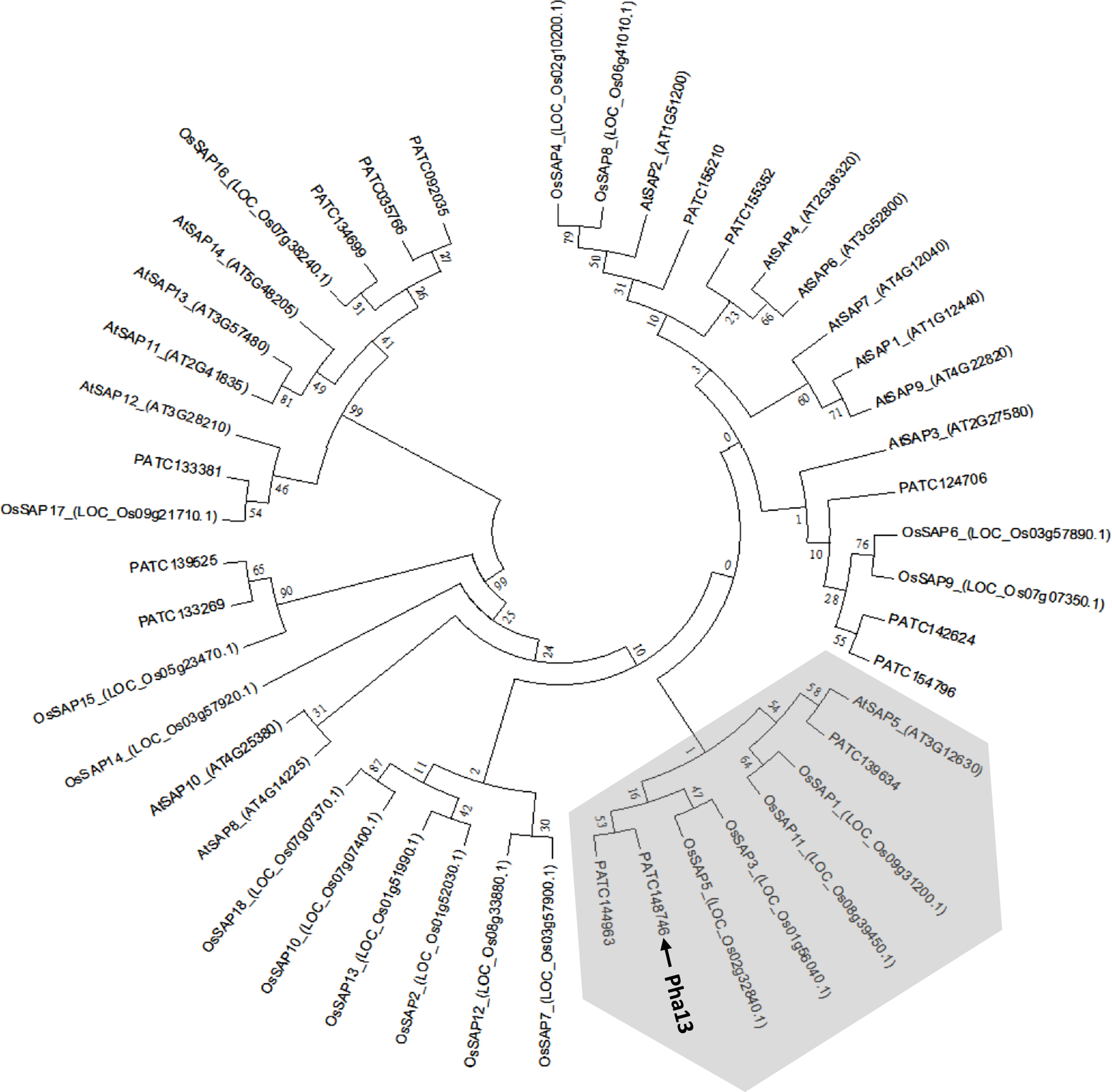

Supplement: S7 Fig — The unrooted phylogenetic tree was constructed using the Clustal X program and neighbor-joining method by MEGA5 with 1000 bootstrap replicates. A20/AN1 zing finger proteins derived from P. aphrodite, A. thaliana, and O. sativa are indicated with the accession number. The branch where Pha13 is located is indicated with a grey hexagonal box. The sequences of SAPs from orchids, A. thaliana and O. sativa were obtained from the websites, Orchidstra database (http://orchidstra2.abrc.sinica.edu.tw/), TAIR (http://www.arabidopsis.org), and the Rice Genome Annotation Project (http://rice.plantbiology.msu.edu), respectively. The accession of each genes is indicated. (TIF) [file ppat.1007288.s011.tif]

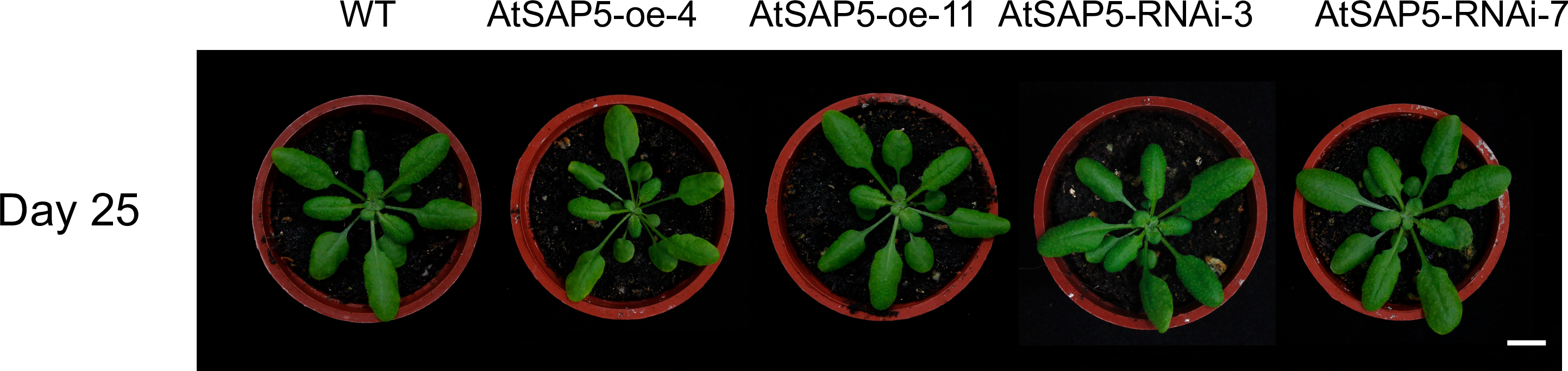

Supplement: S8 Fig — The developmental phenotypes of 25-day–old wild-type (WT, Col-0) Arabidopsis, AtSAP5 overexpression lines (AtSAP5-oe-4 and AtSAP5-oe-11), and RNAi lines (AtSAP5-RNAi-3 and AtSAP5-RNAi-7). Scale bar, 1 cm. (TIF) [file ppat.1007288.s012.tif]

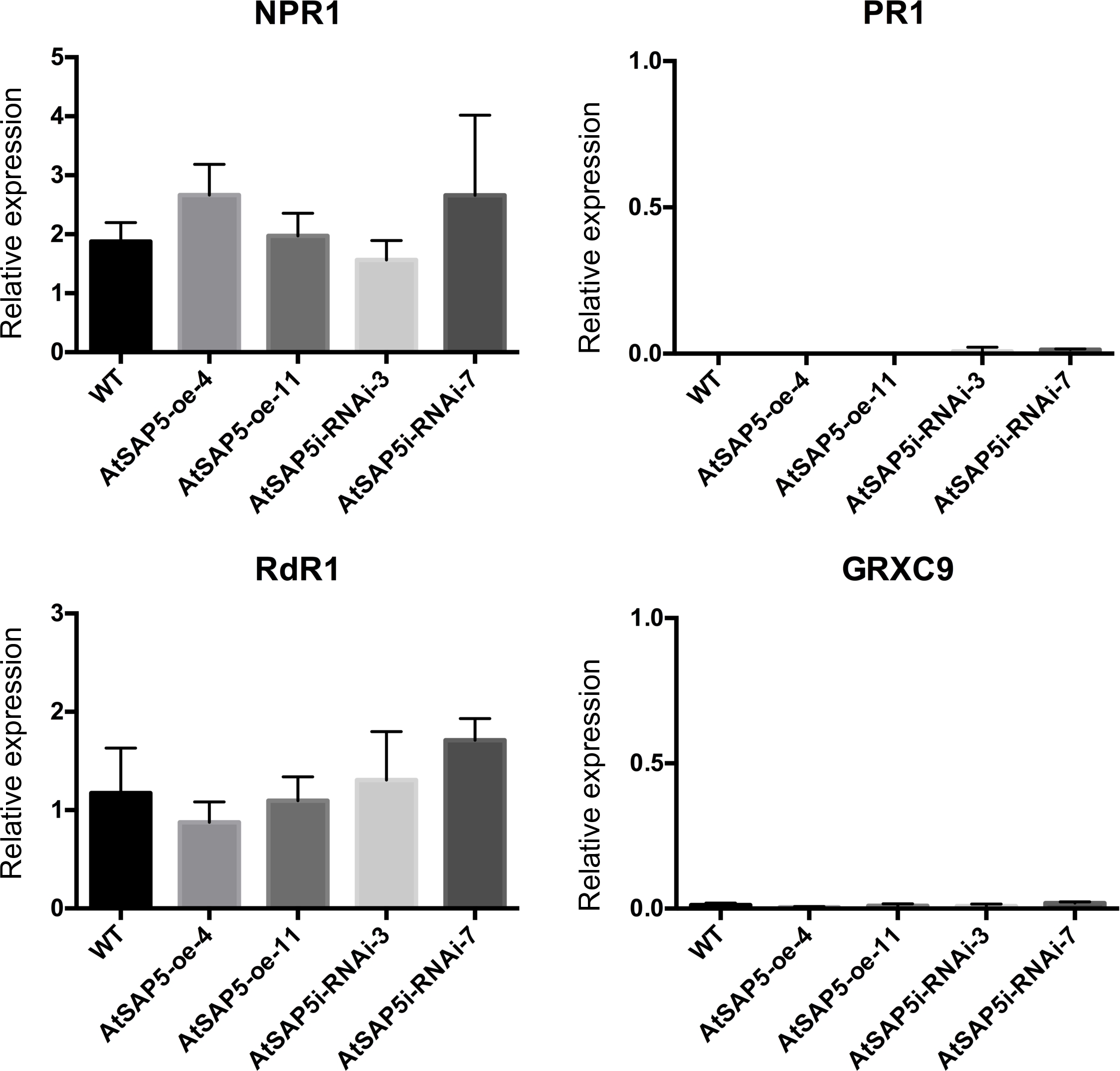

Supplement: S9 Fig — The expression of NPR1, PR1, RdR1, and GRXC9 in WT, AtSAP5 overexpression (AtSAP5-oe-4, AtSAP5-oe-11), and RNAi lines (AtSAP5-RNAi-3 and AtSAP5-RNAi-7) without treatment were analyzed by qRT-PCR. Data represent mean ± SD; n = 3 biological replicates; significant difference was analyzed by Student’s t-test compared to WT. Actin was used as an internal control for normalization. (TIF) [file ppat.1007288.s013.tif]
